# Homeobox protein MSX-1 restricts hepatitis B virus by promoting ubiquitin-independent proteasomal degradation of HBx protein

**DOI:** 10.1371/journal.ppat.1012897

**Published:** 2025-01-30

**Authors:** Qian Qiu, Zihan He, Jing Liu, Huijun Xu, Jinyu Wang, Nannan Liu, Ning Kang, Shaokun Pan, Weien Yu, Zixiang Gao, Shimei Zhang, Yang Yang, Qiang Deng, Youhua Xie, Jiming Zhang, Zhongliang Shen

**Affiliations:** 1 Department of Infectious Diseases, Shanghai Institute of Infectious Diseases and Biosecurity, Shanghai Key Laboratory of Infectious Diseases and Biosafety Emergency Response, National Medical Center for Infectious Diseases, Huashan Hospital, Fudan University, Shanghai, China; 2 Department of Microbiology and Parasitology, School of Basic Medical Sciences, Shanghai Medical College, Key Laboratory of Medical Molecular Virology (Ministry of Education/National Health Commission/Chinese Academy of Medical Sciences), Shanghai Frontiers Science Center of Pathogenic Microbes and Infection, Fudan University, Shanghai, China; 3 Shanghai Key Laboratory of Medical Epigenetics, Institutes of Biomedical Sciences, Shanghai Medical College, Fudan University, Shanghai, China; 4 Children’s Hospital, Fudan University, Shanghai, China; University of Southern California, UNITED STATES OF AMERICA

## Abstract

Hepatitis B virus (HBV) X protein (HBx) is a key factor for regulating viral transcription and replication. We recently characterized homeobox protein MSX-1 (MSX1) as a host restriction factor that inhibits HBV gene expression and genome replication by directly binding to HBV enhancer II/core promoter (EnII/Cp) and suppressing its promoter and enhancer activities. Notably, HBx expression was observed to be repressed more drastically by MSX1 compared to other viral antigens. In this work, we report that in addition to transcriptional repression, MSX1 also post-transcriptionally downregulates HBx protein stability. Mechanistically, MSX1 induces ubiquitin-independent proteasomal degradation of HBx, which is mediated through HBx C-terminal domain. Furthermore, this effect on HBx degradation correlates with MSX1-induced upregulation of DNAJA4 and CRYAB expression. Similar to MSX1, both DNAJA4 and CRYAB promote HBx degradation and repress HBV gene expression and genome replication. In chronic hepatitis B (CHB) patients, immune active phase (IA) is associated with higher intrahepatic expression of MSX1, DNAJA4 and CRYAB, and lower serum HBV markers compared to immune tolerant (IT) phase. Finally, HBV infection is significantly suppressed by MSX1 overexpression in both NTCP-overexpressing cell and humanized liver mouse models. These results demonstrate additional and novel mechanisms of MSX1-mediated repression of HBV, and establish MSX1 as a multi-functional HBV restriction factor with therapeutic potential.

## Author summary

HBx is required to initiate cccDNA transcription and maintain HBV replication, and has been associated with hepatocarcinogenesis. Although previous studies have identified multiple host factors that modulate HBx *in vitro*, regulation of HBx *in vivo* in the context of CHB remains largely elusive. Here, we demonstrate homeobox protein MSX1 degrades HBx via ubiquitin-independent proteasomal pathway, which is closely associated with upregulation of DNAJA4 and CRYAB expression by MSX1 at transcriptional level. DNAJA4- and CRYAB-mediated HBx degradation require an active J domain of DNAJA4 and the interaction between HBx and CRYAB, respectively. At the whole viral genome level, all these host genes could inhibit HBV gene expression and genome replication *in vitro* and *in vivo*. In CHB patients, the mRNA and protein levels of MSX1, DNAJA4 and CRYAB are comparatively higher in IA phase than in IT phase. Based on *in vitro* and *in vivo* infection systems, MSX1 markedly suppresses HBV replication. These results provide insight into the molecular mechanism of how MSX1 suppresses HBV by targeting HBx both *in vitro* and *in vivo*.

## Introduction

Hepatitis B virus (HBV) chronically infects an estimated of 296 million people worldwide [1]. Chronic HBV infection (CHB) is a major risk factor for liver fibrosis, cirrhosis and hepatocellular carcinoma (HCC), causing approximately 0.9 million deaths annually [2]. Nucleos(t)ide analogues and interferon-α (IFNα) are currently used for the treatment of CHB but rarely achieve a functional cure [3]. According to the different viral and host immune status, natural history of CHB is traditionally divided into four phases: immune tolerant (IT), immune active (IA), inactive carrier (IC) and HBV e antigen (HBeAg) negative hepatitis phases [4].

HBV is an enveloped virus with a partially double stranded relax circular DNA (rcDNA) genome and takes humans as its sole natural hosts [5]. In the infected hepatocyte, rcDNA is delivered into the nucleus and converted into covalently closed circular DNA (cccDNA) [5,6]. Viral transcription utilizes cccDNA as the only template and is driven by four promoters, namely core promoter (Cp), surface protein promoters 1 and 2 (Sp1 and Sp2), and X promoter (Xp), to produce 3.5, 2.4, 2.1 and 0.7 kb RNAs, respectively [5,7]. Enhancers I and II (EnI and EnII) overlap with Xp and Cp respectively, and modulate viral transcription in a position- and orientation-independent manner [7]. The 3.5 kb pre-genomic (pg) and preC RNAs function as the translation templates of core protein (HBc) and polymerase (Pol), and secreted HBeAg, respectively [5]. The 2.4/2.1 kb RNAs encode large/middle/small surface antigens (L/M/SHBsAg), while the 0.7 kb RNA encodes X protein (HBx) [5].

The multi-functional virally encoded regulatory protein HBx is required to initiate cccDNA transcription and maintain HBV replication, and has been associated with hepatocarcinogenesis [5,8]. Thus, HBx has become recognized as a potential target for treating CHB and related diseases. Previous studies have identified multiple host factors that modulate HBx expression and/or function *in vitro* [9–11]*.* In contrast, regulation of HBx *in vivo* in the context of CHB remains to be sufficiently studied. Thus, identification of these CHB-related host factors helps understand the interaction between HBV and host *in vivo*, as well as the progression of HBV pathogenesis, to some extent.

Homeobox protein MSX-1 (MSX1) is a DNA-binding transcription factor that plays key roles in embryogenesis and development [12–14], and may also function in tumorigenesis [15,16]. In addition, MSX1 has been shown to participate in innate immune response against Sendai virus and vesicular stomatitis virus [17]. Previously, we reported that elevated intrahepatic MSX1 mRNA levels were associated with better response to IFNα treatment in CHB patients [18]. More recently, we mechanistically confirmed MSX1 as a repressor of HBV gene expression and genome replication, which functions by directly binding to EnII/Cp and inhibiting its promoter and enhancer activities [19]. Notably, compared to other HBV antigens, HBx protein expression was more drastically affected by MSX1 and was nearly abrogated in the presence of overexpressed MSX1 [19]. This suggested possible additional effects of MSX1 on HBx expression. Furthermore, intrahepatic expression levels of MSX1 in IA phase CHB patients were higher than those in IT phase [19], indicating possible participation of MSX1 and related mechanisms in host response against HBV in IA phase. Based on these findings, we hypothesized that in addition to transcriptional repression of EnII/Cp, MSX1 might also downregulate HBx protein expression in a targeted manner, and these diverse effects would make MSX1 a potent restriction factor of HBV *in vitro* and *in vivo* with therapeutic potential.

In this work, we tested the hypothesis and found that MSX1 accelerated ubiquitin-independent proteasomal degradation of HBx. This effect was dependent on the C-terminal domain of HBx, and correlated with transcriptional upregulation of DNAJA4 and CRYAB expression by MSX1. Both DNAJA4 and CRYAB also promoted HBx degradation, which required the J domain of DNAJA4 and interaction between CRYAB and HBx. Similar to MSX1, DNAJA4 and CRYAB inhibited HBV gene expression and genome replication in cell and mouse models of HBV replication, and intrahepatic protein levels of DNAJA4 and CRYAB were higher in IA phase CHB patients compared to IT phase. Finally, therapeutic potential of MSX1 was explored and established using HBV infection models based on NTCP-overexpressing cell line and humanized liver mice.

## Results

### MSX1 post-transcriptionally downregulates HBx expression

Firstly, we reproduced the previously reported potent inhibitory effects of MSX1 on HBV in Huh7 cells transfected with recombinant cccDNA precursor plasmid (prcccDNA) and Cre recombinase overexpression plasmid (pCre) [19,20]. As shown in S1A Fig, viral replication, intracellular HBc and HBx protein levels, and secreted HBeAg and HBsAg levels were all markedly reduced by MSX1 overexpression. Notably, while levels of other viral proteins decreased by 30~50%, HBx was reduced to a nearly undetectable level (S1A Fig). This suggested existence of MSX1-mediated, HBx-specific down-regulation mechanism(s) in addition to MSX1’s binding and repression of EnII/Cp as characterized earlier [19]. Indeed, when each of the seven HBV viral proteins was recombinantly expressed using CMV promoter, only HBx expression was markedly repressed by MSX1 co-transfection (S1B–S1H Fig). This effect was mediated at post-transcriptional level as HBx mRNA levels were unaffected by MSX1 overexpression (S1B Fig, bottom panel).

**Fig 1:**
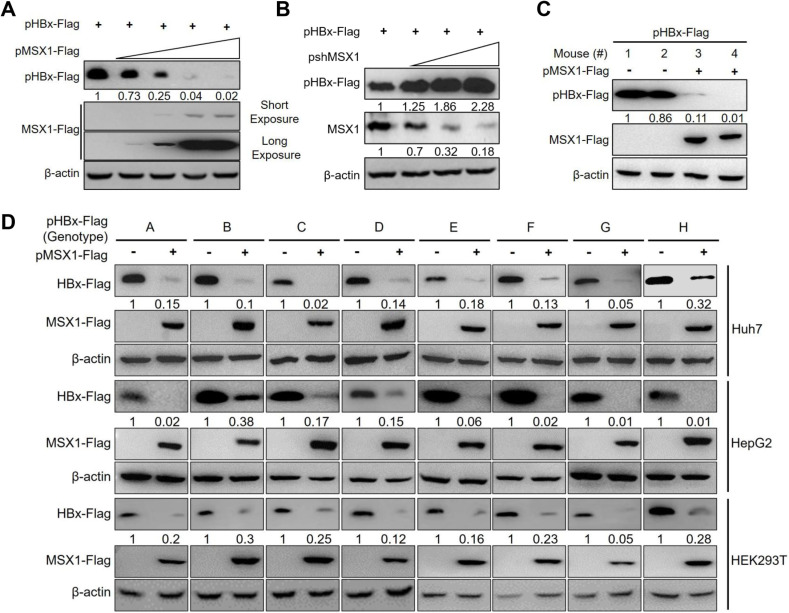
MSX1 reduces HBx protein level *in vitro* and *in vivo.* Huh7 cells were transfected with pHBx-Flag (derived from HBV genotype B) and an increasing amount of pMSX1-Flag (A) or pshMSX1 (B) at a transfection ratio of 1:0, 1:0.5, 1:1, 1:2, 1:3 and 1:0, 1:0.5, 1:1, 1:2, respectively. (C) BALB/c mice were hydrodynamically injected (HDI) with 5 μg of pHBx-Flag and 5 μg of pMSX1-Flag or control plasmid (pCtrl). (D) Huh7, HepG2 and HEK293T cells were transfected with pHBx-Flag of indicated HBV genotype and pMSX1-Flag at a transfection ratio of 1:1. At 3 days post transfection or injection, HBx and exogenous/endogenous MSX1 in cultured cells or mouse livers were determined in Western blot. Protein levels were quantified using densitometry scanning and signal levels in control group were normalized as 1.

To further verify MSX1-mediated down-regulation of HBx protein levels, Huh7 cells were co-transfected with plasmid encoding Flag-tagged HBx (pHBx-Flag) and an increasing amount of plasmid expressing Flag-tagged MSX1 (pMSX1-Flag) or short hairpin RNA targeting MSX1 (pshMSX1). As shown in Fig 1A and 1B, both exogenous Flag-tagged MSX1 and endogenous MSX1 dose-dependently reduced HBx protein levels. In addition, repression of HBx expression was also observed in liver cells of mice hydrodynamically co-injected with pHBx-Flag and pMSX1-Flag (Fig 1C). Furthermore, HBx derived from HBV genotypes A-H were also repressed by MSX1 in Huh7 and HepG2, and to a lesser extent in HEK293T cells (Figs 1D and S2). Clearly, MSX1-mediated downregulation of HBx protein level does not depend on viral genotype or cell type.

### MSX1 promotes ubiquitin-independent proteasomal degradation of HBx

**Fig 2:**
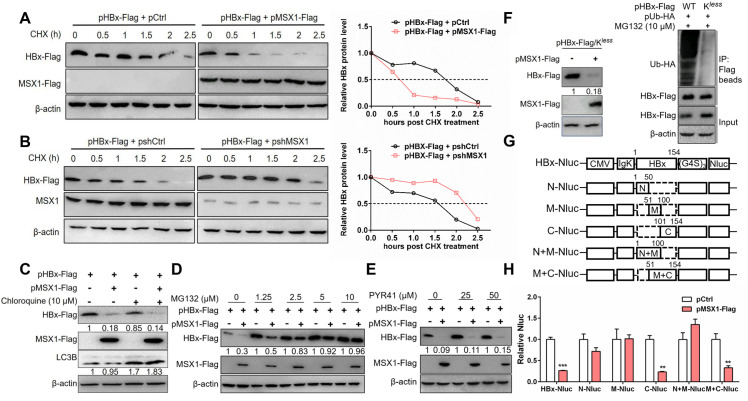
MSX1 promotes ubiquitin-independent proteasomal degradation of HBx. Huh7 cells were transfected with pHBx-Flag plus pMSX1-Flag or pCtrl (A), or plus pshMSX1 or its control plasmid (pshCtrl) (B) at a transfection ratio of 1:1. At 3 days post transfection, cells were treated with CHX (50 μg/ml) for indicated time. (C-E) Huh7 cells were transfected with pHBx-Flag and pMSX1-Flag at a transfection ratio of 1:1. At 60 h post transfection, cells were treated with chloroquine (10 μM) (C), different concentrations of MG132 (D) or PYR41 (E), or left untreated for additional 12 h. HBx and exogenous MSX1 were determined in Western blot using Flag antibody, while endogenous MSX1 and LC3B determined using MSX1 and LC3B antibodies respectively. Protein levels were quantified using densitometry scanning and signal levels in control group were normalized as 1. Quantification of HBx (A and B, right panels) at each time point was normalized to the respective β-actin and then to the HBx level at 0 h. (F) The effects of MSX1 on HBx/K^*less*^ expression in Huh7 cells were examined in Western blot (left). Huh7 cells were transfected with HA-tagged ubiquitin-expressing plasmid (pUb-HA) and pHBx-Flag or pHBx-Flag/K^*less*^. At 60 h post transfection, cells were treated with MG132 for additional 12 h and then subjected to co-immunoprecipitation (Co-IP) assay to compare the ubiquitylation levels between wild type (WT) HBx and HBx/K^*less*^ (right panel). (G) Schematic presentation of the expression cassettes for HBx-fused NanoLuciferase (HBx-Nluc) plasmid and its serial deletion mutants. The HBx-Nluc contains the sequence of HBx, Nluc, and a flexible glycine–serine linker (G4S)3. (G4S)3 is a three-time repeat composed of four glycine (G) and one serine (S) residues. Deleted region within HBx is represented by dashed lines. Numbers denote amino acid positions. (H) Huh7 cultured in 24-well plates were transfected with 0.3 μg of HBx-NLuc or its derived mutants, plus 0.3 μg of pMSX1-Flag or pCtrl. Secreted Nluc was determined using Nluc reporter assays. Group means and SEMs of normalized data were presented and significances calculated using unpaired two-tailed *t* test. **, *P* < 0.01; ***, *P* < 0.001.

To probe mechanism(s) of MSX1-mediated post-transcriptional down-regulation of HBx, Huh7 cells co-transfected with pHBx-Flag and pMSX1-Flag (Fig 2A) or pshMSX1 (Fig 2B) were treated with protein synthesis inhibitor cycloheximide (CHX) to analyze the effects of MSX1 on HBx stability. Overexpression of MSX1 significantly shortened HBx half-life from 1.74 h to 0.67 h, while knockdown of MSX1 extended its half-life from 1.58 h to 2.14 h (Fig 2A and 2B). Next, involvement of lysosome- and proteasome-mediated protein degradation in this process was tested by subjecting transfected Huh7 cells to treatment with lysosome inhibitor chloroquine and proteasome inhibitor MG132. As shown in Fig 2C and 2D, while treatment with 10 μM chloroquine did not alter MSX1’s effect on HBx protein, treatment with increasing concentrations of MG132 mitigated, and abrogated at >=10 μM, accelerated HBx degradation associated with MSX1. Clearly, MSX1 promotes proteasomal, but not lysosomal, degradation of HBx.

As proteasomes catalyze degradation of both poly-ubiquitinated and un-ubiquitinated protein substrates [21,22], we went on to probe whether MSX1-promoted HBx degradation depends on HBx ubiquitination. Treatment of HBx and MSX1 co-transfected Huh7 cells with PYR41, an inhibitor of ubiquitin activating enzyme E1 and consequently cell-wide ubiquitination, failed to counteract the effect of MSX1 on HBx degradation (Fig 2E). On the other hand, an HBx mutant with all lysine (K) residues mutated to arginine (R) (HBx/K^*less*^) virtually lost the ability of being poly-ubiquitinated, but was still susceptible to MSX1-promoted degradation (Fig 2F). Collectively, these results demonstrated MSX1 promotes ubiquitin-independent proteasomal degradation of HBx.

To delineate domain(s) of HBx involved in MSX1-promoted degradation, we used our previously established reporter system [23] and expressed full-length and deletion mutant HBx as secreted proteins fused to N-terminal NanoLuc luciferase (Nluc). NLuc activities in culture media were used as a surrogate indicator of HBx-NLuc protein level. N-terminal (N, amino acid (aa) 1–50), middle (M, aa 51–100) and C-terminal (C, aa 101–154) domains of HBx, as well as N+M and M+C domains of HBx, were thus expressed fused to NLuc (Fig 2G). Significant reduction of NLuc activities by MSX1 co-transfection was observed for full-length HBx, and also for all deletion constructs containing C-terminal domain of HBx (Fig 2H). This result suggested that MSX1-promoted degradation very likely targets HBx C-terminal domain.

### Screening and identification of MSX1-regulated host genes associated with promoting HBx degradation

**Fig 3:**
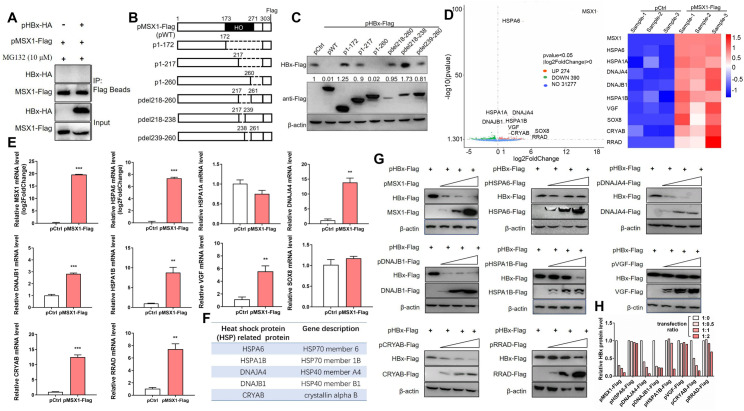
Screening and identification of MSX1’s downstream effector genes associated with promoting HBx degradation. (A) Huh7 cells were transfected with pMSX1-Flag and HA-tagged HBx-expressing plasmid (pHBx-HA) or its control plasmid at a transfection ratio of 1:1. At 60 h post transfection, cells were treated with MG132 for additional 12 h. Interaction between MSX1 and HBx was analyzed using Co-IP assay. (B) Schematic representation of pMSX1-Flag and its serial deletion mutants. Deleted region within MSX1 is represented by dashed lines, and numbers denote amino acid positions. (C) Western blot was performed to analyze the effects of MSX1 and its deletion mutants on HBx expression in Huh7 cells. (D) Huh7 cells were transfected with pMSX1-Flag or pCtrl in triplicates and then subjected to transcriptome sequencing analysis. Volcano plot (left) and heat map (right) were presented with top 10 upregulated DEGs indicated. (E) RT-qrtPCR assay was performed on pMSX-Flag- or pCtrl-transfected Huh7 cells to determine the mRNA expression levels of indicated genes. (F) 5 of the 7 confirmed MSX1-upregulated genes belong to heat shock protein (HSP) families. (G) Huh7 cells were transfected with pHBx-Flag and an increasing amount of Flag-tagged MSX1, HSPA6, DNAJA4, DNAJB1, HSPA1B, VGF, CRYAB, or RRAD overexpression plasmid at a transection ratio of 1:0, 1:0.5, 1:1, 1:2. Western blot was utilized to analyze the effects of exogenously expressed genes on HBx. (C and H) HBx protein levels were quantified using densitometry scanning and signal levels in control group were normalized as 1. Group means and SEMs of normalized data were presented and significances calculated using unpaired two-tailed *t* test. **, *P* < 0.01; ***, *P* < 0.001.

MSX1 is a transcription factor that binds DNA through the internal homeodomain (aa 173–271) [24,25], and has not been previously shown to affect degradation of other protein(s) directly. Co-immunoprecipitation (Co-IP) assay indeed failed to show any physical association between MSX1 and HBx (Fig 3A), whereas MSX1 mutants containing deletion in the DNA-binding homeobox (HO) domain, particularly aa 218–260, displayed reduced or no effects on HBx degradation (Fig 3B and 3C). It is therefore plausible that MSX1 promotes HBx degradation indirectly by transcriptionally modulating the expression of other factors.

Potential MSX1-regulated genes were then screened through transcriptome sequencing of Huh7 cells transfected with pMSX1-Flag or pCtrl. MSX1 overexpression was associated with significant changes in the transcription levels of 664 genes, 274 upregulated and 390 downregulated, among 31941 human genes analyzed (Fig 3D and S1 Table). From these differentially expressed genes (DEGs), the top 10 upregulated ones including MSX1 (as positive control), HSPA6, HSPA1A, DNAJA4, DNAJB1, HSPA1B, VGF, SOX8, CRYAB and RRAD (Fig 3D) were selected for further analysis. RT-qrtPCR results showed that, except for HSPA1A and SOX8, MSX1 overexpression markedly increased transcription of the other 7 genes (Fig 3E). Interestingly, 5 of the 7 confirmed MSX1-upregulated genes are members of heat shock protein (HSP) 20/40/70 families (Fig 3F). The effects of 7 MSX1-upregulated DEGs on HBx were then tested, and co-transfection of exogenous DNAJA4, DNAJB1 and CRYAB reduced HBx protein levels in a dose-dependent manner similar to MSX1 in both Huh7 (Fig 3G and 3H) and HepG2 cells (S3 Fig). In HepG2 cells, however, only transcription of DNAJA4 and CRYAB, but not DNAJB1, was upregulated by MSX1 overexpression (S4A–S4D Fig). At high dosages, HSPA1B expression was also associated with lower HBx protein levels (Fig 3G and 3H). These results suggested the possibility that MSX1-activated expression of HSP proteins, especially DNAJA4, DNAJB1 and CRYAB, might participate in promoting HBx degradation.

### MSX1 promotes HBx degradation by transcriptionally activating DNAJA4 and CRYAB expression

**Fig 4:**
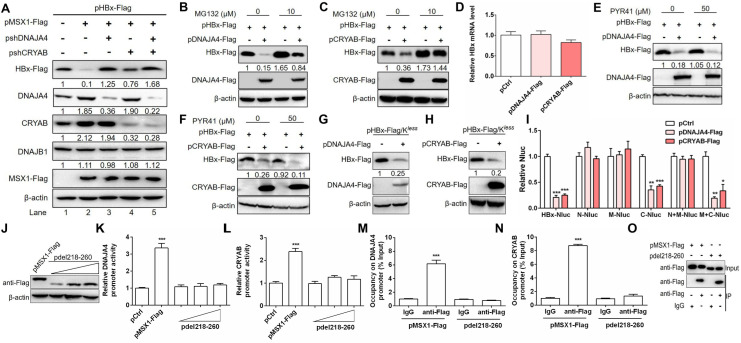
MSX1 promotes HBx degradation by transcriptionally activating DNAJA4 and CRYAB expression. Huh7 cells cultured in 12-well plates were transfected with 0.5 µg of pHBx-Flag and 0.5 µg of pMSX1-Flag or pCtrl, plus 0.5 µg of pshDNAJA4 or pshCRYAB or both (A). Huh7 cells were transfected with pHBx-Flag and pDNAJA4-Flag (B and E), or pCRYAB-Flag (C and F) or pCtrl at a transfection ratio of 1:1. At 60 h post transfection, cells were treated with MG132 (B and C) or PYR41 (E and F) for additional 12 h. At 3 days post transfection, HBx and exogenous/endogenous host proteins were determined in Western blot. (D) Huh 7 cells were transfected with pHBx-Flag and pDNAJA4-Flag, or pCRYAB-Flag or pCtrl at a transfection ratio of 1:1. At 3 days post transfection, cells were subjected to RT-qrtPCR to analyze HBx mRNA levels. The effects of exogenous DNAJA4 (G) and CRYAB (H) on HBx/K^*less*^ expression in Huh7 cells were examined in Western blot. (I) Huh7 cultured in 24-well plates were transfected with 0.3 μg of HBx-NLuc or its serial deletion mutants, plus 0.3 μg of pDNAJA4-Flag, pCRYAB-Flag or pCtrl. Secreted Nluc was determined using Nluc reporter assays. Protein levels were quantified using densitometry scanning and signal levels in control group were normalized as 1. Huh7 cells cultured in 24-well plates were transfected with 0.3 µg of DNAJA4 promoter reporter plasmid (J and K) or CRYAB promoter reporter plasmid (L), 0.1 µg of Renilla luciferase reporter plasmid (pRL-TK), plus 0. 3 µg of pMSX1-Flag or pCtrl, or 0.3, 0.6, 0.9 µg of pdel218–260. At 2 days post transfection, wild type and mutant MSX1 were determined in Western blot (J) while DNAJA4 and CRYAB promoter activities determined using dual-luciferase reporter assay (K and L). Huh7 cells cultured in 6-cm dishes were transfected with 2 µg of pMSX1-Flag or 6 µg of pdel218–260. At 3 days post transfection, cells were subjected to ChIP assay, and sonicated DNA immunoprecipitated by Flag antibody or mouse control IgG was quantitated in qrtPCR using specific primers targeting DNAJA4 promoter (M) and CRYAB promoter (N), and presented as percentage of input. Immunoprecipitated wild type and mutant MSX1 were determined using Western blot (O). Group means and SEMs of normalized values were presented and significances calculated using unpaired two-tailed *t* test. *, *P* < 0.05; **, *P* < 0.01; ***, *P* < 0.001.

To establish functional involvement of MSX1-upregulated genes in promoting HBx degradation, we first tested effects of endogenous DNAJA4, DNAJB1 and CRYAB levels on HBx protein. Huh7 cells were co-transfected with pHBx-Flag and pMSX1-Flag, along with shDNAJA4-expressing plasmid (pshDNAJA4), shCRYAB-expressing plasmid (pshCRYAB) or both. As shown in Fig 4A, MSX1 overexpression upregulated endogenous DNAJA4 and CRYAB, but not DNAJB1, protein expression (lanes 1–2). Similar results were obtained in HepG2 cells (S4E Fig). Knockdown of endogenous DNAJA4, CRYAB or both through RNA interference mitigated or abrogated MSX1-associated HBx degradation (Fig 4A). Furthermore, similar to MSX1 (Figs 1 and S1B), both exogenous DNAJA4 and CRYAB downregulated HBx protein expression without affecting HBx mRNA (Fig 4B–4D), which were counteracted by treatment with MG132 (Figs 4B, 4C and S5) but not PYR41 (Fig 4E and 4F), remained effective on ubiquitination-deficient HBx (Fig 4G and 4H), and apparently also depended on the C-terminal domain of HBx (Fig 4I). These results strongly supported DNAJA4 and CRYAB as MSX1’s downstream effectors in promoting HBx degradation via an ubiquitin-independent proteasome pathway.

As the DNA-binding homeodomain of MSX1 was critical for MSX1-promoted HBx degradation (Fig 3B and 3C), wild type and del218–260 mutant (aa 218–260 in homeodomain deleted) MSX1 were compared for their abilities to upregulate DNAJA4 and CRYAB expression. Dual-luciferase reporter assay showed that both DNAJA4 and CRYAB promoters were activated by overexpression of MSX1, but not the del218–260 mutant (Fig 4J–4L). Accordingly, chromatin immunoprecipitation (ChIP) analysis confirmed occupancy of both endogenous and exogenous MSX1, but not the del218–260 mutant, on both promoters (Figs 4M–4O and S6A–S6C). Conversely, knockdown of endogenous MSX1 decreased the activities of DNAJA4 and CRYAB promoters in reporter assay (S6D and S6E Fig). These data confirmed DNAJA4 and CRYAB as direct targets of MSX1-mediated transcriptional activation.

### DNAJA4 and CRYAB promote HBx degradation through an active J domain and association with HBx C-terminal domain, respectively

**Fig 5:**
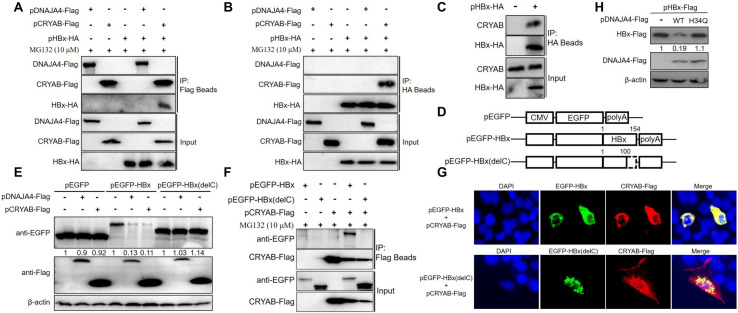
DNAJA4 and CRYAB promote HBx degradation through an active J domain and association with HBx C-terminal domain, respectively. Huh7 cells were transfected with pDNAJA4-Flag, pCRYAB-Flag or pCtrl, together with or without pHBx-HA co-transfection. At 60 h post transfection, cells were treated with MG132 for additional 12 h. Interaction between DNAJA4/CRYAB and HBx was determined via Co-IP assay using anti-Flag (A) or anti-HA (B) beads. (C) The interaction between endogenous CRYAB and HBx was determined in pHBx-HA transfected Huh7 cells using Co-IP assay. (D) Schematic presentation of plasmids expressing EGFP, EGFP-HBx or EGFP-HBx(delC). Deleted region within HBx is represented by dashed lines. Numbers denote amino acid positions. (E) Huh7 cells were transfected with pEGFP, pEGFP-HBx or pEGFP-HBx(delC), plus pDNAJA4-Flag, pCRYAB-Flag or pCtrl at a transfection ratio of 1:1. At 3 days post transfection, proteins were analyzed via Western blot using anti-EGFP or anti-Flag antibody. (F) The interaction between EGFP-HBx/EGFP-HBx(delC) and exogenous CRYAB was analyzed using Co-IP assay as similarly done in A. (G) Huh7 cells were co-transfected with pCRYAB-Flag and pEGFP-HBx or pEGFP-HBx(delC). At 2 days post transfection, intracellular distribution of exogenous CRYAB and EGFP-HBx or EGFP-HBx(delC) was analyzed in immunofluorescence assay. (H) Comparison of the effects of wild type DNAJA4 and its mutant with H34Q substitution on HBx expression in Huh7 cells. Protein levels were quantified using densitometry scanning and signal levels in control group were normalized as 1.

Since previous studies have identified host proteins that interact HBx to modulate its proteasomal degradation [9,26,27], we performed reciprocal Co-IP assays using Huh7 cells co-transfected with pHBx-HA and pDNAJA4-Flag or pCRYAB-Flag. Co-IP of HBx with exogenous CRYAB, but not exogenous DNAJA4, was observed (Fig 5A and 5B), and association between HBx and endogenous CRYAB was also observed (Fig 5C). Next, we analyzed the correlation between CRYAB-mediated HBx degradation and their interaction. Wild type and mutant HBx lacking the C-terminal domain were fused downstream of enhanced green fluorescent protein (EGFP) to generate plasmids expressing fusion proteins EGFP-HBx and EGFP-HBx(delC) respectively (Fig 5D). Western blot revealed that CRYAB markedly decreased the protein level of EGFP-HBx, but not EGFP or EGFP-HBx(delC) (Fig 5E), consistent with results obtained using the NLuc reporter system (Fig 4I). Accordingly, CRYAB co-immunoprecipitated with EGFP-HBx, but not EGFP-HBx(delC) (Fig 5F), while immunofluorescence assay revealed EGFP-HBx, but not EGFP-HBx(delC), altered the originally diffuse subcellular localization of exogenous CRYAB and caused marked co-localization (Fig 5G). Collectively, these results suggested that CRYAB mediates HBx degradation through a direct or indirect interaction with the latter’s C-terminal domain.

DNAJA4 and DNAJB1 are both members of the HSP40/DnaJ family, which features a highly conserved N-terminal J domain that mediates the interaction with HSP70 and regulates its ATPase activity [28,29]. The three amino acid HPD motif in J domain is highly conserved and crucial for J domain activity, with a single mutation of H to Q resulting in inactivation of J domain [30]. Previous studies showed that DNAJB1 promotes HBx degradation by proteasome in a J domain-dependent manner [31,32]. Therefore, we investigated whether J domain was essential for DNAJA4-mediated HBx degradation. As shown in Fig 5H, DNAJA4 with H34Q mutation in J domain indeed lost the ability to promote HBx degradation, suggesting a dependence on functional J domain.

### DNAJA4 and CRYAB HBx-dependently inhibit HBV gene expression and genome replication *in vitro* and *in vivo*

**Fig 6:**
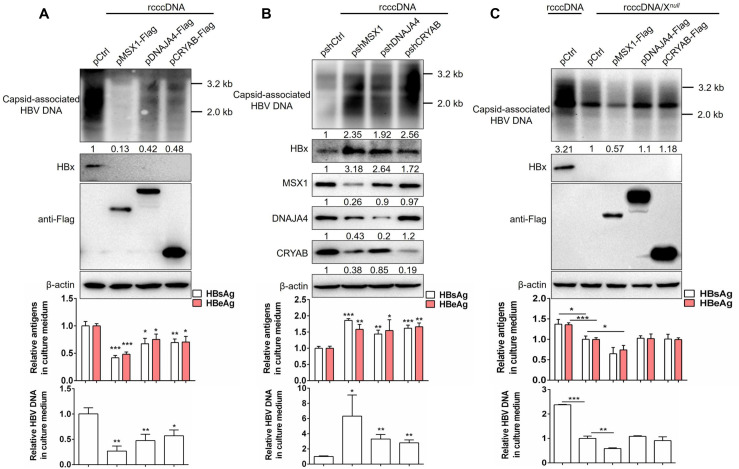
DNAJA4 and CRYAB HBx-dependently inhibit HBV gene expression and genome replication *in vitro.* Huh7 cells cultured in 6-well plates were transfected with 1 µg of prcccDNA (A and B) or prcccDNA/X^*null*^ (C) and 1 µg of pCre, plus 1 µg of pMSX1-Flag, pDNAJA4-Flag, pCRYAB-Flag or pCtrl (A and C), or plus 1 µg of pshMSX1, pshDNAJA4, pshCRYAB or pshCtrl (B). At 3 days post transfection, intracellular HBV replication, HBx and exogenous/endogenous host proteins were analyzed using Southern and Western blots, respectively. Secreted HBV antigens (HBsAg and HBeAg) and supernatant HBV DNA were assayed using ELISA and qrtPCR, respectively. Viral replication and protein levels were quantified using densitometry scanning and normalized using respective control as 1. Group means and SEMs of normalized values were presented and significances calculated using unpaired two-tailed *t* test. *, *P* < 0.05; **, *P* < 0.01; ***, *P* < 0.001.

Since HBx plays a critical role in HBV gene expression and genome replication [8], we tested the effects of MSX1-activated DNAJA4 and CRYAB expression and consequent HBx degradation on HBV life cycle. Huh7 cells were transfected with prcccDNA and pCre, along with pMSX1-Flag, pDNAJA4-Flag, pCRYAB-Flag or pCtrl (Fig 6A), or pshMSX1, pshDNAJA4, pshCRYAB or pshCtrl (Fig 6B). Similar to MSX1, overexpression of DNAJA4 or CRYAB markedly repressed HBV replication and production of secreted antigens and progeny HBV DNA, albeit to a lesser extent (Fig 6A). By contrast, HBx protein was similarly decreased to levels below detection limit by MSX1, DNAJA4 or CRYAB (Fig 6A). Conversely, knockdown of either MSX1, DNAJA4 or CRYAB resulted in increased HBx protein levels, HBV antigen secretion, genome replication and progeny virus production (Fig 6B). Knockdown of endogenous MSX1 also expectedly repressed expression of endogenous DNAJA4 and CRYAB, and produced more pronounced effects on HBV (Fig 6B). Both exogenous and endogenous MSX1, DNAJA4 and CRYAB dose-dependently inhibited HBx expression (S7 Fig). Meanwhile, MSX1-mediated repression of HBV was not due to the hepatoma cell damage as overexpression or knockdown of MSX1 did not affect the cell viability (S8 Fig). Next, when mutant prcccDNA with HBx ORF obliterated (prcccDNA/X^*null*^) was used instead of wild type prcccDNA, neither DNAJA4 nor CRYAB overexpression displayed any effect on viral gene expression or genome replication, which were still markedly repressed by MSX1 overexpression (Fig 6C). This is consistent with MSX1-mediated repression of EnII/Cp [19], and lack of effects of DNAJA4 or CRYAB on HBV promoter activities (S9 Fig). Lastly, when pCMV1.1HBV replicon plasmid containing 1.1-fold HBV genome under the control of CMV promoter instead of EnII/Cp was used, MSX1 overexpression markedly inhibited the production of secreted antigens production and genome replication, and reduced HBx expression to an undetectable level (S10 Fig). Collectively, these results demonstrated that HBx protein degradation mediated by DNAJA4 and CRYAB results in repression of HBV, while activation of DNAJA4 and CRYAB expression constitutes another mechanism of MSX1-mediated restriction of HBV, in addition to inhibition of EnII/Cp [19].

Effects of MSX1 and its effector genes on HBV were then evaluated *in vivo.* BALB/c mice were co-injected with prcccDNA and pCre, together with pMSX1-Flag, pDNAJA4-Flag, pCRYAB-Flag or pCtrl through hydrodynamic injection via tail vein. At 3 days post injection, serum and liver tissue samples were collected. As shown in S11 Fig, compared to pCtrl injection group, serum HBV antigens (HBeAg and HBsAg), intracellular HBV replication and capsid levels decreased in the other 3 groups. pMSX1-Flag injected mice displayed more pronounced inhibition of HBV compared to pDNAJA4-Flag or pCRYAB-Flag injected mice. These data were consistent with results obtained *in vitro* (Fig 6A).

### Profile of expression of MSX1 and its effector genes in IT and IA phases of CHB

**Fig 7:**
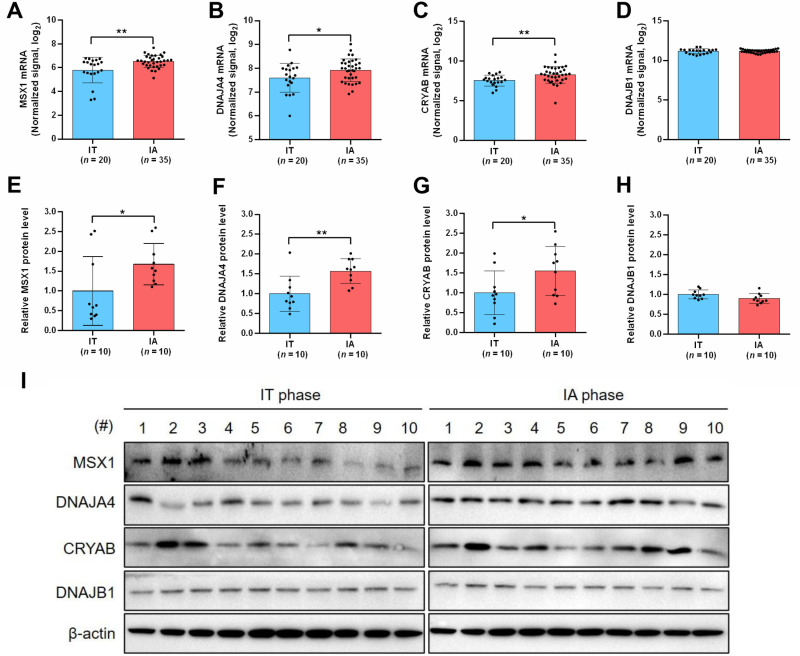
Comparison of expression of MSX1 and its effector genes in CHB patients at IT phase *vs.* IA phase. Liver biopsy samples collected from patients in IT and IA phases were subjected to RNA microarray analysis in our previous study [33] and intrahepatic MSX1 (A), DNAJA4 (B), CRYAB (C) and DNAJB1 (D) RNA levels were analyzed with group size indicated (*n*). Biopsy samples from 20 IT and IA patients (S2 Table) were assayed for protein expression of above genes using Western blot (I). Protein levels were quantified using densitometry scanning (E-H). Group means and SEMs were presented and significances calculated using unpaired two-tailed *t*-test. *, *P* < 0.05; **, *P* < 0.01.

In our previous study using liver biopsy specimens from CHB patients in IT and IA phases, RNA microarray analysis identified MSX1, DNAJA4 and CRYAB, but not DNAJB1, as upregulated DEGs with elevated mRNA levels in IA phase samples compared to IT phase samples (Fig 7A–7D) [33]. To assess protein expression of these genes, a subset of biopsy samples derived from 10 IT and 10 IA patients were subjected to Western blot analysis. The IA phase patients had lower serum HBV DNA and elevated ALT levels compared to those in IT phase (S2 Table and S12A Fig). Intrahepatic protein levels of MSX1, DNAJA4 and CRYAB were higher in IA phase samples, while DNAJB1 protein levels were comparable (Fig 7E–7I). In addition, a positive correlation between MSX1 and CRAYB protein levels could be observed (S12B and S12C Fig). Given MSX1-, DNAJA4- and CRYAB-mediated potent antiviral effects (Figs 6, S7 and S11), elevated expression of these genes in IA phase could be expected to contribute towards host control of HBV activities.

### MSX1 inhibits HBV gene expression and genome replication in infected HepG2-NTCP cells and human liver-chimeric mice

To test the effects of MSX1 on early steps of HBV life cycle, HepG2 cells stably expressing HBV receptor NTCP (HepG2-NTCP) were first transduced with lentivirus expressing Flag-tagged MSX1 (Lenti-MSX1-Fag) or expressing shMSX1 (Lenti-shMSX1). At 3 days post transduction, cells were infected with HBV and 7 days later, cells and culture media were collected for analysis (S13A Fig). As shown in S13B Fig, exogenous MSX1 overexpression markedly decreased intracellular HBV replication, secreted HBsAg and HBeAg production, and repressed HBx expression to undetectable level. More importantly, nuclear cccDNA levels were reduced 6–7 folds by MSX1 overexpression. Conversely, knockdown of endogenous MSX1 prior to HBV infection resulted in elevated HBV gene expression and genome replication, as well as higher cccDNA levels (S13C Fig).

Secondly, we compared the effects of MSX1 and its downstream effector proteins on HBV in HepG2-NTCP cells. As shown in S14 Fig, both exogenous and endogenous MSX1, DNAJA4, and CRYAB markedly restricted HBV gene expression and genome replication, similar to the results from rcccDNA transfected cells and hydrodynamically injected mice (Figs 6A, 6B and S11). In all these systems, MSX1 expectedly showed more potent inhibitory effects compared to DNAJA4 and CRYAB.

**Fig 8:**
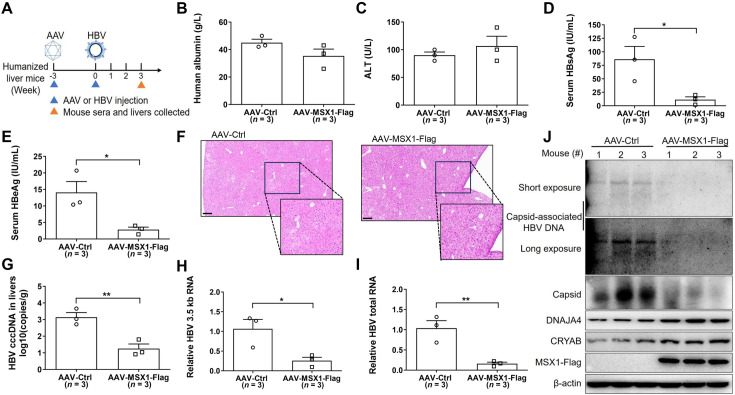
MSX1 restricts HBV gene expression and replication in a humanized liver mouse model of HBV infection. (A) Schematic description of the experiments in human liver-chimeric mice. Mice were injected with AAV-MSX1-Flag or AAV-Ctrl via tail vein and each group contained 3 mice. At 3 weeks post AAV injection, mice were injected with HBV, and 3 weeks later serum and liver tissue samples collected. Serum human albumin (B), ALT (C), HBsAg (D) and HBeAg (E) were analyzed in commercial quantitative assays. (F) H&E staining was performed on liver tissues. HBV cccDNA (G), 3.5 kb RNA (H) and total viral RNA (I) in livers were analyzed using qrtPCR. (J) Intracellular HBV replication, endogenous DNAJA4/CRYAB and exogenous MSX1 were analyzed using Southern and Western blots, respectively. Capsid levels were determined using native agarose gel electrophoresis followed by Western blot. Group means and SEMs of normalized values were presented and significances calculated using unpaired two-tailed *t* test. *, *P* < 0.05; **, *P* < 0.01. Scale bars: 200 μm.

Finally, to test the effects of MSX1 on HBV infection of human hepatocytes *in vivo*, human liver-chimeric mice were first injected with adeno-associated virus expressing Flag-tagged MSX1 (AAV-MSX1-Flag) or control virus (AAV-Ctrl), and 3 weeks later, infected with HBV. At 3 weeks post HBV infection, mouse sera and liver tissues were collected (Fig 8A). AAV-MSX1-Flag-injected mice (*n* = 3) displayed much lower levels of serum HBsAg and HBeAg compared to AAV-Ctrl-injected mice (*n* = 3), while levels of human albumin and alanine aminotransferase (ALT) in serum were comparable in both groups (Fig 8B–8E). Hematoxylin-eosin (H&E) staining showed no obvious immune infiltration in both groups either (Fig 8F). Notably, cccDNA levels in livers of AAV-MSX1-Flag-injected mice were significantly reduced (Fig 8G), along with markedly lower intracellular HBV transcription (HBV 3.5 kb RNA and total RNA levels), capsid and capsid-associated HBV progeny DNA levels (Fig 8H–8J). Furthermore, intrahepatic protein levels of endogenous DNAJA4 and CRYAB were higher in mice receiving AAV-MSX1-Flag injection (Fig 8J). Taken together, these results established MSX1 as a potent host restriction factor of HBV *in vitro* and *in vivo*.

## Discussion

HBx is essential for activating and maintaining viral transcription from cccDNA via diverse mechanisms [5,8]. On the other hand, HBx also affects expression of multiple host genes and has been associated with the pathogenesis of HBV-related HCC [8]. Thus, HBx has become a promising target for the treatment of CHB and related liver diseases [2,5,8]. In this work, we show that host homeobox protein MSX1 promotes HBx protein degradation (Figs 1 and 2) by activating the expression of HSP proteins DNAJA4 and CRYAB (Figs 3–5). MSX1 is a transcriptional factor that binds to cognate site(s) in DNA via its HO domain and regulates the expression of target genes crucial for embryogenesis and development [13,24,25]. It is interesting that in Huh7 cells, a majority (5 of 7) of the top MSX1-upregulated genes that were identified by transcriptome sequencing and confirmed by RT-qrtPCR, encode proteins of HSP families (Fig 3). In both Huh7 and HepG2 cells, MSX1-activation of DNAJA4 and CRYAB expression were also confirmed at protein level (Figs 4 and S4). Whether such a pattern could also be observed in cells derived from other tissue and organ types, and whether it is mechanistically related to MSX1-associated phenotypes warrant further study. It is also worth noting that although ChIP analysis demonstrated occupancy of MSX1, but not the HO-deficient MSX1 mutant, on DNAJA4 and CRYAB promoters (Fig 4), mechanism of MSX1-mediated transcriptional activation of these genes remains to be addressed in more detail.

DNAJA4 is a member of the HSP40/DnaJ family of proteins that are essential components of the Hsp40/Hsp70 chaperone system, responsible for substrate recruitment and stimulation of Hsp70 ATPase activity through the conserved J domain [28,29]. Hsp40-triggered ATP hydrolysis causes conformational changes in Hsp70, and the resultant high-substrate-affinity ADP state trapped the substrate for subsequent sequestration, refolding or degradation [28,29]. Two HPS40 proteins, DNAJB1 and DNAJA3, were previously shown to facilitate HBx degradation via the proteasome pathway [31,32]. Data presented here demonstrate that DNAJA4 promotes ubiquitin-independent proteasomal degradation of HBx, which requires a functionally active J domain of DNAJA4 (Figs 4 and 5). Given that at high dosages, a Hsp70 protein (HSPA1B) was also associated with decreased HBx protein levels (Fig 3E–3H), it is likely that Hsp40/Hsp70 chaperone system is involved in recruiting HBx for ubiquitin-independent degradation through proteasome. Since no association between HBx and DNAJA4 was observed in Co-IP assay (Fig 5), HBx recognition and subsequent recruitment to Hsp70 by DNAJA4 could be a fast process, with only transient association between HBx and DNAJA4. Alternatively, other HSP40 family member(s) could be involved. Additional studies are clearly required to elucidate the underlying mechanisms.

CRYAB, also known as HSPB5, belongs to the small HSP (sHSP or HSP20) family, which is characterized by an internal α-crystallin domain and intrinsic ATP-independent chaperone-like properties [34]. Hsp20 members form oligomers that interact with partially unfolded proteins and sequester the latter as soluble complexes, and protect cells against various stress stimuli by preventing irreversible protein aggregation [35]. The effect of CRYAB on HBx protein degradation as demonstrated in this work (Fig 5) represent the first report of an Hsp20 that modulates HBx protein stability. An earlier study identified interaction between CRYAB and 20S proteasome component C8/α7 [36]. Whether such an interaction participates in CRYAB-promoted HBx degradation by proteasome should be a focus of follow-up work.

Association of CRYAB with HBx in Co-IP and their co-localization in immunofluorescence possibly indicate more direct involvement of CRYAB in HBx protein homeostasis compared to DNAJA4, which displays no detectable association with HBx in Co-IP (Fig 5). Meanwhile, as both DNAJA4- and CRYAB-promoted degradation apparently act through the C-terminal domain of HBx (Figs 4 and 5), similarity and overlapping of underlying mechanisms are very likely. This is additionally supported by the fact that MSX1-activated CRYAB expression could not compensate for knockdown of DNAJA4 in facilitating HBx degradation, and *vice versa* (Fig 4A). In other words, adequate DNAJA4 and CRYAB levels appear to be both required for efficient HBx degradation.

Although either DNAJA4 or CRYAB alone was capable of suppressing HBV gene expression and genome replication, MSX1 displayed more potent HBV-repressing effects (Figs 6 and S14), both as a result of simultaneous activation of DNAJA4 and CRYAB expression by MSX1 (Fig 4), and also due to MSX1-mediated repression of EnII/Cp promoter and enhancer activities [19]. Such orthogonal repression mechanisms targeting different aspects of HBV life cycle makes MSX1 an attractive candidate for the therapeutic intervention in CHB. Higher intrahepatic MSX1 expression in CHB patients in IA phase compared to IT phase apparently links MSX1 to host restriction and control attempts targeting HBV, which further suggested its therapeutic potential. Indeed, artificially engendered MSX1 overexpression significantly repressed viral activities in HBV-infected cultured cells *in vitro* (S13 Fig), and human hepatocytes in chimeric liver mice (Fig 8). Additional work in more relevant animal models of CHB is required to fully characterize MSX1’s HBV-repressing effects and its safety profile *in vivo*.

In summary, results presented in this and our previous work collectively establish intrahepatic MSX1 as a novel restriction factor of HBV that acts through multiple mechanisms to mediate potent repression of HBV both *in vitro* and *in vivo*.

## Materials and methods

### Ethics statement

The study received permission (2020–375) from the Ethics Committee of Huashan Hospital, Fudan University. All the study participants were enrolled in Huashan Hospital, and the written informed consent was obtained from all enrollees. Mouse procedures were approved by the Animal Ethics Committee of School of Basic Medical Sciences, Fudan University. The approval number was 2021JS-063.

### Cells and chemical compounds

Human hepatocellular carcinoma cells (Huh7 and HepG2), HepG2 with stably integrated inducible HBV genome (HepAD38), or HBV receptor NTCP (HepG2-NTCP) [6] and human embryonic kidney 293T cells (HEK293T) were cultured as previously described [19]. Chemical compounds chloroquine, MG132 and PYR41 were purchased from TOPSCIENCE (China), and cycloheximide was from Selleck (China).

### Plasmids, cell transfection and luciferase reporter assays

The HBx open reading frame (ORF) was cloned from HBV genotype A-H strains and inserted downstream of Flag tag in pcDNA3.0-N-Flag (Invitrogen, China) to obtain corresponding pHBx-Flag. The GenBank Accession Numbers of genotype A, B, D, E, F, G, and H strains are AP007263.1, KR152339, V01460, X75664.1, X75663, GU565217 and AB516393.1, respectively. The genotype C strain has not been submitted to GenBank, but has been used in our previous studies and others [37]. ORFs of HBx (derived from HBV genotype D) and ubiquitin were inserted downstream of HA tag in pcDNA3.0-N-HA (Invitrogen) to obtain pHBx-HA and pUb-HA, respectively. Flag-tagged HBc, Pol, L/M/SHBs and His-tagged HBe overexpression plasmids have been described previously [38].

ORF of MSX1 (Gene ID 4487) was cloned upstream of Flag tag in pCMV-C-Flag (pCtrl) (Beyotime, China) and His tag in pCMV-C-His (Beyotime) to generate plasmids pMSX1-Flag and pMSX1-His respectively. Flag tagged HSPA6 (Gene ID 3310), DNAJA4 (Gene ID 55466), DNAJB1 (Gene ID 3337), HSPA1B (Gene ID 3304), VGF (Gene ID 7425), CRYAB (Gene ID 1410) and RRAD (Gene ID 6236) overexpression plasmids were similarly constructed as pMSX1-Flag. The HBx-fused Nanoluciferaese reporter plasmid (HBx-Nluc) has been described previously [23]. HBx ORF derived from HBV genotype B was cloned downstream of EGFP in pCMV-EGFP-C2 (pEGFP, Invitrogen) to generate pEGFP-HBx. Serial deletion mutant plasmids based on HBx-Nluc, pMSX1-Flag, pEGFP-HBx were constructed using site-directed mutagenesis (Toyobo, Japan) as indicated in Figs 2G, 3B and 5D, respectively. DNAJA 4 promoter sequences (nt −2169 to −170) or CRYAB promoter sequences (nt −2198 to −199) were inserted into pGL3-basic (Promega, China) to create corresponding luciferase reporter plasmid. Nucleotides were numbered using first nucleotide of respective ORF as + 1 and the immediate upstream nucleotide as −1. The reporter plasmids of four HBV promoters (Cp, Sp1, Sp2 and Xp) have been described previously [38].

Recombinant cccDNA plasmid (prcccDNA, GenBank Accession Number: V01460), Cre recombinase overexpression plasmid (pCre), and pCMV1.1HBV replicon plasmid have been described previously [20,39]. HBx ORF in prcccDNA was obliterated by mutating CAA to TAA at 8th amino acid to create prcccDNA/X^*null*^.

For knockdown of endogenous genes expression, DNA fragments encoding short hairpin RNA (shRNA) targeting MSX1, DNAJA4 and CRYAB were cloned downstream of U6 promoter in pLKO.1 (pshCtrl, Addgene, USA) to create pshMSX1, pshDNAJA4 and pshCRYAB, respectively. The corresponding target sequences of RNA interference are “CCCGAGAGGACCCCGTGGATGCAGA”, “ATCCGTGAGAAGAAGATTATC” and “TGTGATTGAGGTGCATGGAAA”, respectively.

Cell transfection was carried out using TurboFect Transfection Reagent (Thermo Fisher, China). Dual Luciferase Report System and Nano-Glo Luciferase System (Promega) were used for analysis of promoter activities and secreted Nluc levels in culture medium respectively, according to the manufacturers’ instructions.

### Cell viability

Cell viability was assessed using a cell counting kit-8 (CCK8) (Dojindo, Japan) according to the manufacturer’s instruction.

### Transcriptome sequencing

Huh7 cells cultured in 12-well plates were transfected with 1 μg of pMSX1-Flag or pCtrl in triplicates. At 3 days post transfection, total RNA extracted from cultured cells was subjected to library construction and deep sequencing on Illumina NovaSeq 6000 by Novogene Biotechnology Co. Ltd. (Shanghai, China). FPKM, namely fragments per kilobase of transcript per million mapped reads, was used to estimate expression levels of host gens. Differential expression analysis of two groups was performed using the DESeq2 R package (1.20.0). Differentially expressed genes (DEGs) with a fold change > 2 and a false discovery rate (FDR) < 0.05 were considered as candidate DEGs and presented in S1 Table.

### HBV antigens and nucleic acids analysis

HBV antigens (HBsAg and HBeAg) in culture medium and mouse serum were measured using ELISA Kits (Kehua, China) and commercial quantitative assays (Labway, China), respectively. HBV capsid-associated DNA in cytoplasm and cccDNA in extrachromosomal nuclear DNA were extracted as previously described [19,38]. For Southern blot, DNA samples were subjected to electrophoresis in 1% agarose and transferred onto nylon membrane. The membrane was hybridized with DIG-labeled full-length HBV probe, and signals developed using DIG Luminescent Detection Kit (Roche, Germany).

### Western blot

Total cell lysates were prepared from cultured cells, human and mouse liver samples using SDS lysis buffer (Beyotime). Mouse liver tissues were lysed in TBS buffer (10 mM Tris-HCl at pH = 7.0, 150 mM NaCl) with 0.5% NP-40 followed by centrifugation. Viral capsids were separated by electrophoresis in 1% agarose gel and then blotted onto nitrocellulose for western blot analysis. Western blot was performed as previously described [19,38] using following antibodies: anti-Flag (1:10,000) and anti-β-actin (1:10,000) from Sigma; anti-MSX1 (1:3,000, #4787S), anti-LC3B (1:1,000, #3868S) and anti-HA (1:1,000, #3724S) from Cell Signaling Technology; anti-DNAJB1 (1:1,000, RK05643), anti-DNAJA4 (1:1,000, A21155) and anti-CRYAB (1:1,000, A13696) from Abclonal; anti-EGFP from Abmart (1:3000, M20004); anti-HBc (1:10,000, Denmark) from DAKO. Mouse anti-HBx mAb 2A7 (1:1,000) has been previously described [40,41].

### Chromatin immunoprecipitation (ChIP) and co-immunoprecipitation (Co-IP)

ChIP assay was performed using SimpleChIP Plus Enzymatic Chromatin IP Kit (#9005, Cell Signaling Technology) according to the manufacturer’s instruction. MSX1 and Flag antibodies were used as listed above for Western blot.

Co-IP was performed as previously described [19] using antibodies as listed above for Western blot. Anti-Flag Magnetic Beads (M8823) and anti-HA Agarose Beads (M20031M) were purchased from Sigma and Abmart, respectively.

### Immunofluorescence (IF) assay

IF assay performed on cultured cells was described previously [19,42]. Briefly, cells were firstly washed with PBS, fixed in 4% paraformaldehyde for 30 min, and washed with PBS. The cells were blocked with 3% BSA in PBST buffer (PBS plus 0.5% Triton X-100) for 30 min, incubated with primary anti-Flag antibody (1:1,000, as listed for Western blot) diluted in 3% BSA in PBST buffer for 2 h at room temperature, and finally stained with Alexa Fluor 546-labeled secondary antibody (1:1,000, Life Technologies, USA) for 1 h at room temperature. Cell nuclei were stained with 4,6-diamidino-2-phenylindole (DAPI, Sangon, China) and observed through an AMG EVOS fluorescence microscope (Mill Creek, USA).

### Virus preparation and infection

Recombinant lentiviruses expressing Flag-tagged MSX1 (Lenti-MSX1-Flag) and shMSX1 (Lenti-shMSX1) have been obtained as previously described [19]. Lenti-DNAJA4 and Lenti-CRYAB were prepared as similarly done for Lenti-MSX1.

HBV virions were obtained by concentration of supernatants from HepAD38 cells and sera from HBV transgenic mice (GenBank Accession Number: AF305422.1), and then used for infection of HepG2-NTCP cells and human liver-chimeric mice (Hu-URG mice), respectively. *In vitro* infection assay was performed at 1,000:1 multiplicity of infection (MOI) as previously described [19,38]. Both HBV transgenic mice and Hu-URG mice were purchased from Vitalstar (Beijing, China) and have been used in prior works [43,44].

Recombinant serotype 8 adeno-associated virus expressing Flag-tagged human MSX1 (AAV-MSX1-Flag) and its control virus (AAV-Ctrl) were purchased from Obio Technology, China.

### Patient liver samples

A total of 55 CHB patients were enrolled, with 20 patients in IT phase and 35 patients in IA phase. Liver biopsy samples were subjected to RNA extraction and microarray analysis using Affymetrix Human Genome U133 Plus 2.0 Array as described in a previous study [33]. Biopsy samples from 20 IT and IA patients were also assayed for MSX1, DNAJA4, CRYAB and DNAJB1 expression using Western blot. The clinical and virological information of CHB patients at IT and IA phases were presented in S2 Table.

### Quantitative real-time PCR for mRNA and HBV cccDNA

For mRNA quantification, total cellular RNA was subjected to reverse transcription using PrimeScriptRT Reagent Kit (TaKaRa, China) to obtain cDNA. cDNA and HBV cccDNA were quantified in real-time PCR using SYBR Premix Ex Taq II Kit (TaKaRa). The primer sequences are listed in S3 Table.

### Mouse work

Male BALB/c mice aged 6–8 weeks were purchased from Shanghai Slac Laboratory Animal Co. Ltd. For evaluating the effects of MSX1 on HBx expression *in vivo*, mice were hydrodynamically injected (HDI) with 5 μg of pHBx-Flag and 5 μg of pMSX1-Flag or pCtrl via tail vein. For evaluating the effects of MSX1 and its downstream effector genes on HBV *in vivo*, mice were injected with 10 μg of prcccDNA and 10 μg of pCre, plus 10 μg of pMSX1-Flag, pDNAJA4-Flag, pCRYAB-Flag or pCtrl via HDI method. At 3 days post injection, mouse serum samples were collected through retro-orbital sinus bleeding, and liver tissues taken after sacrifice by CO_2_ treatment.

For determining the effects of MSX1 on HBV infection *in vivo*, humanized liver mice were injected with 2×10^11^ genome equivalent (geq) of AAV-MSX1-Flag or AAV-Ctrl via tail vein. At 3 weeks post AAV injection, mice were injected with concentrated sera containing 1×10^8^ geq of HBV from HBV transgenic mice via tail vein, and 3 weeks later serum and liver tissue samples collected for further analysis. Serum human albumin and ALT were measured using commercial quantitative assays (Labway, China). H&E staining was performed on liver tissues to examine immune infiltration.

### Statistical analysis

Group means and standard errors (SEM) from independently repeated experiments were indicated and subjected to unpaired two-tailed *t*-test. A *P*-value < 0.05 was considered statistically significant. GraphPad 6.0 was used for plotting and statistical tests.

## Supporting information

S1 Fig Effects of MSX1 overexpression on HBV at the whole and sub viral genome levels. (A) Huh7 cells cultured in 6-well plates were transfected with 1μg of prcccDNA and 1 μg of pCre, plus 1μg of pMSX1-Flag or control plasmid (pCtrl). At 3 days post transfection, intracellular HBV replication and viral proteins (HBc and HBx) were assayed by Southern and Western blots, respectively. Exogenous MSX1 was determined in Western blot using Flag antibody and secreted antigens (HBsAg and HBeAg) were examined using ELISA. Huh7 cells cultured in 12-well plates were transfected with 0.5 μg of pHBx-Flag (B), pHBc-Flag (C), pHBe-His (D), pSHBs-Flag (E), pMHBs-Flag (F), pLHBs-Flag (G) or pPol-Flag (H), and 0.5 μg of pMSX1-Flag (B-E), pMSX1-His (F-H) or pCtrl. At 3 days post transfection, viral proteins and exogenous MSX1 were determined in Western blot using Flag or His antibody (B, top panel, C-H) while HBx mRNA levels determined in qrtPCR (B, bottom panel). HBV replication and protein levels were quantified using densitometry scanning and signal levels in control group were normalized as 1. Group means and SEMs of normalized values were presented and significances calculated using unpaired two-tailed *t* test. *, *P* < 0.05; ***, *P* < 0.001.
(TIF)

S2 Fig MSX1 reduces HBx protein level *in vitro.
* Huh7, HepG2 and HEK293T cells were transfected with pHBx-Flag of indicated HBV genotype and pMSX1-Flag at a transfection ratio of 1:1. At 3 days post transfection, HBx and exogenous MSX1 were determined in Western blot using Flag antibody. The experiments were repeated twice as shown in (A) and (B). HBx protein levels were quantified using densitometry scanning and signal levels in control group normalized as 1. (E) Analysis of MSX1’s effects on HBx protein expression of indicated genotype in hepatoma and HEK 293T cells based on three repeats (Figs 1D, S2A and S2B). HBx protein level in MSX1-transfected group was presented after normalized to control group. Group means and SEMs were presented and significances calculated using unpaired two-tailed *t* test. **, *P* < 0.01.
(TIF)

S3 Fig Overexpression of DNAJA4, CRYAB or DNAJB1 reduces HBx expression in HepG2 cells. HepG2 cells were co-transfected with pHBx-Flag and an increasing amount of pMSX1-Flag (A), pDNAJA4-Flag (B), pCRYAB-Flag (C) or pDNAJB1-Flag (D) at a transfection ratio of 1:0, 1:0.5, 1:1, 1:2. At 3 days post transfection, the expression levels of HBx and exogenous genes were determined using Flag antibody in Western blot. (E) HBx protein levels were quantified using densitometry scanning and signal levels in control group were normalized as 1.
(TIF)

S4 Fig MSX1 upregulates the expression of DNAJA4 and CRYAB expression in HepG2 cells. RT-qrtPCR (A-D) and Western blot assays (E) were performed on pMSX1-Flag- and pCtrl-transfected HepG2 cells to determine the effects of MSX1 on endogenous DNAJA4, CRYAB and DNAJB1 expression at mRNA and protein levels respectively. Protein levels were quantified using densitometry scanning and signal levels in control group were normalized as 1 (E). Group means and SEMs of normalized values were presented and significances calculated using unpaired two-tailed *t* test. *, *P* < 0.05; **, *P* < 0.01; ***, *P* < 0.001.
(TIF)

S5 Fig Analysis of the effects of proteasome inhibitor MG132 treatment on DNAJA4-promoted HBx degradation. Huh7 cells were transfected with pHBx-Flag and pDNAJA4-Flag or pCtrl at a transfection ratio of 1:1. At 48 h post transfection, cells were treated with different concentrations of MG132 or left untreated for additional 24 h. HBx and exogenous DNAJA4 were determined in Western blot using Flag antibody. HBx protein levels were quantified using densitometry scanning and signal levels in pCtrl-transfected group were normalized as 1.
(TIF)

S6 Fig Endogenous MSX1 activates promoter activities of both DNAJA4 and CRYAB and binds to both promoters. Huh7 cells were subjected to ChIP assay, and sonicated DNA immunoprecipitated by anti-MSX1 or rabbit control IgG was quantitated in qrtPCR using specific primers targeting DNAJA4 promoter (A) or CRYAB promoter (B) and indicated as percentage of input. Immunoprecipitated MSX1 was determined in Western blot (C). Huh7 cells cultured in 24-well plates were transfected with 0.3 μg of DNAJA4 promoter reporter plasmid (D) or CRYAB promoter reporter plasmid (E), plus 0.1 μg of pRL-TK and 0.3 μg of pshMSX1 or pshCtrl. At 2 days post transfection, cells were lysed and promoter activities were measured using dual-luciferase reporter assay. Group means and SEMs of normalized values were presented and significances calculated using unpaired two-tailed *t* test. **, *P* < 0.01; ***, *P* < 0.001.
(TIF)

S7 Fig MSX1, DNAJA4 and CRYAB does-dependently reduce HBx protein. Huh7 cells cultured in 12-well plates were transfected with 0.5 μg of prcccDNA and 0.5 μg of pCre, plus 0.1 or 0.5 μg of pMSX1-Flag (A), pDNAJA4-Flag (B) or pCRYAB-Flag (C), or plus 0.25 or 0.5 μg of pshMSX1 (D), pshDNAJA4 (E) or pshCRYAB (F). At 3 days post transfection, HBx, exogenous and endogenous MSX1, DNAJA4 and CRYAB were determined using Western blot. HBx protein levels were quantified using densitometry scanning and signal levels in control group were normalized as 1.
(TIF)

S8 Fig Effects of overexpression or knockdown of MSX1 on cell viability. Hepatoma cells (Huh7 and HepG2) cultured in 96-well plates were transfected with indicated amounts of pMSX1-Flag or pCtrl (A-B), pshMSX1 or pshCtrl (C-D), or left untreated. Cell viability was detected at day 1, 2 and 3 post transfection using CCK-8 assays. Group means and SEMs within transfection group were presented.
(TIF)

S9 Fig Analysis of the effects of MSX1, DNAJA4 and CRYAB on HBV promoter activities. Huh7 cells cultured in 24-well plates were transfected with 0.3 µg of Cp, Sp1, Sp2, or Xp reporter plasmid, 0.3 µg of pMSX1-Flag, pDNAJA4-Flag, pCRYAB-Flag or pCtrl, and 0.1 µg of pRL-TK. At 48 h post transfection, cells were lysed and subjected to dual-luciferase reporter assay. Group means and SEMs of normalized firefly versus Renilla luciferase activity ratios were presented, and significances calculated using unpaired two-tailed *t*-test. ***, *P* < 0.001.
(TIF)

S10 Fig MSX1 inhibits CMV-driven HBV gene expression and genome replication. Huh7 cells cultured in 12-well plates were transfected with 1 µg of pCMV1.1HBV and 1 µg of pMSX1-Flag or pCtrl. At 3 days post transfection, intracellular HBV replication, HBx and exogenous MSX1 were analyzed using Southern and Western blots, respectively. Secreted HBV antigens (HBsAg and HBeAg) were assayed using ELISA. Group means and SEMs of normalized values were presented and significances calculated using unpaired two-tailed *t* test. **, *P* < 0.01.
(TIF)

S11 Fig MSX1, DNAJA4 and CRYAB suppress HBV gene expression and genome replication *in vivo.
* BALB/c mice were co-injected with prcccDNA and pCre, plus pMSX1-Flag, pDNAJA4-Flag, pCRYAB-Flag or pCtrl through HDI method. At 3 days post injection, serum and liver tissues samples were taken. Serum HBsAg (A) and HBeAg (B) were analyzed using commercial quantitative assays with group size indicated (*n*). (C) Intracellular HBV replication and exogenous genes expression were analyzed using Southern and Western blots, respectively. Capsid levels were determined using native agarose gel electrophoresis followed by Western blot. Group means and SEMs were presented and significances calculated using unpaired two-tailed *t* test. *, *P* < 0.05; **, *P* < 0.01; ***, *P* < 0.001.
(TIF)

S12 Fig Correlation analysis of intrahepatic protein expression of MSX1, DNAJA4 and CRYAB. (A) Comparison of serum HBV DNA between IT and IA phases with group size (*n*) indicated. The correlation analysis between MSX1 and DNAJA4 (B) or CRYAB (C) at protein expression levels based on the data from Fig 7E, 7F, 7G and 7I.
(TIF)

S13 Fig MSX1 restricts HBV gene expression and replication in HepG2-NTCP cells. HepG2-NTCP cells were first transduced with Lenti-MSX1-Flag or Lenti-Ctrl (A and B), or Lenti-shMSX1 or Lenti-shCtrl (A and C), and 3 days later, infected with HBV at 1000 geq/cell. Culture media were changed at indicated time points. At 7 days post HBV infection, culture media and cells were collected for further analysis. HBsAg and HBeAg in culture media were analyzed using ELISA. Intracellular HBV replication and cccDNA were analyzed using Southern blot and qrtPCR, respectively. HBx and exogenous/endogenous MSX1 were analyzed using Western blot. Viral replication and protein levels were quantified using densitometry scanning and signal levels in control group were normalized as 1. Group means and SEMs were presented and significances calculated using unpaired two-tailed *t* test. *, *P* < 0.05; **, *P* < 0.01; ***, *P* < 0.001.
(TIF)

S14 Fig Analysis of the effects of MSX1, DNAJA4 and CRYAB on HBV in HepG2-NTCP cells. HepG2-NTCP cells in 24-well pates were transfected with 1 μg of pMSX1-Flag, pDNAJA4-Flag, pCRYAB-Flag or pCtrl (A), or transduced with Lenti-shMSX1, Lenti-DNAJA4, Lenti-CRYAB or Lenti-shCtrl (B), and 3 days later, infected with HBV at 1000 geq/cell. Culture media were changed at day 1, 2 and 4 post HBV infection. At 7 days post HBV infection, HBsAg and HBeAg in culture media were analyzed using ELISA. Intracellular HBV replication was analyzed using qrtPCR. Exogenous/endogenous MSX1, DNAJA4 and CRYAB were analyzed using Western blot. Viral replication and serum antigens in control group were normalized as 1. Group means and SEMs were presented and significances calculated using unpaired two-tailed *t* test. *, *P* < 0.05; **, *P* < 0.01; ***, *P* < 0.001.
(TIF)

S1 Table Detailed information of DEGs post MSX1 overexpression. (XLSX)

S2 Table Clinical and virological information of CHB patients in IT and IA phases. (DOCX)

S3 Table Primer sequences used for RT-qrtPCR or qrtPCR analysis. (DOCX)

## References

[1] Jeng W-J, Papatheodoridis GV, Lok ASF. Hepatitis B. Lancet. 2023;401(10381):1039–52. doi: 10.1016/S0140-6736(22)01468-4 36774930

[2] Liang TJ. Hepatitis B: the virus and disease. Hepatology. 2009;49(5 Suppl):S13–21. doi: 10.1002/hep.22881 19399811 PMC2809016

[3] Fanning GC, Zoulim F, Hou J, Bertoletti A. Therapeutic strategies for hepatitis B virus infection: towards a cure. Nat Rev Drug Discov. 2019;18(11):827–44. doi: 10.1038/s41573-019-0037-0 31455905

[4] Terrault NA, Bzowej NH, Chang K-M, Hwang JP, Jonas MM, Murad MH, et al. AASLD guidelines for treatment of chronic hepatitis B. Hepatology. 2016;63(1):261–83. doi: 10.1002/hep.28156 26566064 PMC5987259

[5] Seeger C, Mason WS. Molecular biology of hepatitis B virus infection. Virology. 2015;479–480:672–86. doi: 10.1016/j.virol.2015.02.031 25759099 PMC4424072

[6] Yan H, Zhong G, Xu G, He W, Jing Z, Gao Z, et al. Sodium taurocholate cotransporting polypeptide is a functional receptor for human hepatitis B and D virus. Elife. 2012;1:e00049. doi: 10.7554/eLife.00049 23150796 PMC3485615

[7] Quasdorff M, Protzer U. Control of hepatitis B virus at the level of transcription. J Viral Hepat. 2010;17(8):527–36. doi: 10.1111/j.1365-2893.2010.01315.x 20546497

[8] Levrero M, Zucman-Rossi J. Mechanisms of HBV-induced hepatocellular carcinoma. J Hepatol. 2016;64(1 Suppl):S84–101. doi: 10.1016/j.jhep.2016.02.021 27084040

[9] Wu Q, Zhang L, Xu X, Zhang Y, Shi J, Lin X, et al. Hepatitis B virus X protein is stabilized by the deubiquitinating enzyme VCPIP1 in a Ubiquitin-independent manner by recruiting the 26S proteasome subunit PSMC3. J Virol. 2022;96(13):e0061122. doi: 10.1128/jvi.00611-22 35695579 PMC9278118

[10] Kouwaki T, Okamoto T, Ito A, Sugiyama Y, Yamashita K, Suzuki T, et al. Hepatocyte factor JMJD5 regulates hepatitis B virus replication through interaction with HBx. J Virol. 2016;90(7):3530–42. doi: 10.1128/JVI.02776-15 26792738 PMC4794694

[11] Ariffianto A, Deng L, Abe T, Matsui C, Ito M, Ryo A, et al. Oxidative stress sensor Keap1 recognizes HBx protein to activate the Nrf2/ARE signaling pathway, thereby inhibiting hepatitis B virus replication. J Virol. 2023;97(10):e0128723. doi: 10.1128/jvi.01287-23 37800948 PMC10617466

[12] Xie H, Cherrington BD, Meadows JD, Witham EA, Mellon PL. Msx1 homeodomain protein represses the αGSU and GnRH receptor genes during gonadotrope development. Mol Endocrinol. 2013;27(3):422–36. doi: 10.1210/me.2012-1289 23371388 PMC3589667

[13] Sarapura VD, Strouth HL, Gordon DF, Wood WM, Ridgway EC. Msx1 is present in thyrotropic cells and binds to a consensus site on the glycoprotein hormone alpha-subunit promoter. Mol Endocrinol. 1997;11(12):1782–94. doi: 10.1210/mend.11.12.0015 9369446

[14] Park K-S, Kim KK, Kim KE. Msx1 homeodomain transcription factor and TATA-binding protein interact to repress the expression of the glycoprotein hormone α subunit gene. Biochem Biophys Res Commun. 2015;468(1–2):326–30. doi: 10.1016/j.bbrc.2015.10.102 26505791

[15] Katoh M. Multi‑layered prevention and treatment of chronic inflammation, organ fibrosis and cancer associated with canonical WNT/β‑catenin signaling activation (Review). Int J Mol Med. 2018;42(2):713–25. doi: 10.3892/ijmm.2018.3689 29786110 PMC6034925

[16] Heppt MV, Wang JX, Hristova DM, Wei Z, Li L, Evans B, et al. MSX1-induced neural crest-like reprogramming promotes melanoma progression. J Invest Dermatol. 2018;138(1):141–9. doi: 10.1016/j.jid.2017.05.038 ; PMCID: PMC5902795. 28927893 PMC5902795

[17] Chen L-T, Hu M-M, Xu Z-S, Liu Y, Shu H-B. MSX1 modulates RLR-mediated innate antiviral signaling by facilitating assembly of TBK1-associated complexes. J Immunol. 2016;197(1):199–207. doi: 10.4049/jimmunol.1600039 27194789

[18] Li N, Yu K, Dong M, Wang J, Yang F, Zhu H, et al. Intrahepatic transcriptomics reveals gene signatures in chronic hepatitis B patients responded to interferon therapy. Emerg Microbes Infect. 2022;11(1):1876–89. doi: 10.1080/22221751.2022.2100831 35815389 PMC9336496

[19] Shen Z, Zhang S, Gao Z, Yu X, Wang J, Pan S, et al. Intrahepatic homeobox protein MSX-1 is a novel host restriction factor of hepatitis B virus. J Virol. 2024;98(2):e0134523. doi: 10.1128/jvi.01345-23 38226815 PMC10878074

[20] Qi Z, Li G, Hu H, Yang C, Zhang X, Leng Q, et al. Recombinant covalently closed circular hepatitis B virus DNA induces prolonged viral persistence in immunocompetent mice. J Virol. 2014;88(14):8045–56. doi: 10.1128/JVI.01024-14 24807718 PMC4097776

[21] Varshavsky A. The Ubiquitin system, autophagy, and regulated protein degradation. Annu Rev Biochem. 2017;86:123–8. doi: 10.1146/annurev-biochem-061516-044859 28654326

[22] Bialek W, Collawn JF, Bartoszewski R. Ubiquitin-dependent and independent proteasomal degradation in host-pathogen interactions. Molecules. 2023;28(18):6740. doi: 10.3390/molecules28186740 37764516 PMC10536765

[23] Gu C, Tao S, Hu K, Ming L, Luo M, Guo H, et al. Establishment of an in vitro reporter system for screening HBx-targeting molecules. Acta Biochim Biophys Sin (Shanghai). 2019;51(4):431–40. doi: 10.1093/abbs/gmz016 30811522

[24] Pellerin I, Schnabel C, Catron KM, Abate C. Hox proteins have different affinities for a consensus DNA site that correlate with the positions of their genes on the hox cluster. Mol Cell Biol. 1994;14(7):4532–45. doi: 10.1128/mcb.14.7.4532-4545.1994 7911971 PMC358825

[25] Hoffmann HM, Catron KM, van Wijnen AJ, McCabe LR, Lian JB, Stein GS, et al. Transcriptional control of the tissue-specific, developmentally regulated osteocalcin gene requires a binding motif for the Msx family of homeodomain proteins. Proc Natl Acad Sci U S A. 1994;91(26):12887–91. doi: 10.1073/pnas.91.26.12887 7809141 PMC45545

[26] Tan G, Yi Z, Song H, Xu F, Li F, Aliyari R, et al. Type-I-IFN-stimulated gene TRIM5γ inhibits HBV replication by promoting HBx degradation. Cell Rep. 2019;29(11):3551–3563.e3. doi: 10.1016/j.celrep.2019.11.041 31825835 PMC6996557

[27] Liu C, Zhao K, Chen Y, Yao Y, Tang J, Wang J, et al. Mitochondrial glycerol-3-phosphate dehydrogenase restricts HBV replication via the TRIM28-mediated degradation of HBx. J Virol. 2023;97(5):e0058023. doi: 10.1128/jvi.00580-23 37166302 PMC10231258

[28] Ohtsuka K, Hata M. Molecular chaperone function of mammalian Hsp70 and Hsp40—a review. Int J Hyperthermia. 2000;16(3):231–45. doi: 10.1080/026567300285259 10830586

[29] Cheetham ME, Caplan AJ. Structure, function and evolution of DnaJ: conservation and adaptation of chaperone function. Cell Stress Chaperones. 1998;3(1):28–36. doi: 10.1379/1466-1268(1998)003<0028:sfaeod>2.3.co;2 9585179 PMC312945

[30] Qian YQ, Patel D, Hartl FU, McColl DJ. Nuclear magnetic resonance solution structure of the human Hsp40 (HDJ-1) J-domain. J Mol Biol. 1996;260(2):224–35. doi: 10.1006/jmbi.1996.0394 8764402

[31] Sohn S-Y, Kim S-B, Kim J, Ahn B-Y. Negative regulation of hepatitis B virus replication by cellular Hsp40/DnaJ proteins through destabilization of viral core and X proteins. J Gen Virol. 2006;87(Pt 7):1883–91. doi: 10.1099/vir.0.81684-0 16760390

[32] Sohn S-Y, Kim J-H, Baek K-W, Ryu W-S, Ahn B-Y. Turnover of hepatitis B virus X protein is facilitated by Hdj1, a human Hsp40/DnaJ protein. Biochem Biophys Res Commun. 2006;347(3):764–8. doi: 10.1016/j.bbrc.2006.06.158 16842747

[33] Liu H, Li F, Zhang X, Yu J, Wang J, Jia J, et al. Differentially expressed intrahepatic genes contribute to control of hepatitis B virus replication in the inactive carrier phase. J Infect Dis. 2018;217(7):1044–54. doi: 10.1093/infdis/jix683 29300924

[34] Edwards HV, Cameron RT, Baillie GS. The emerging role of HSP20 as a multifunctional protective agent. Cell Signal. 2011;23(9):1447–54. doi: 10.1016/j.cellsig.2011.05.009 21616144

[35] Haslbeck M, Vierling E. A first line of stress defense: small heat shock proteins and their function in protein homeostasis. J Mol Biol. 2015;427(7):1537–48. doi: 10.1016/j.jmb.2015.02.002 25681016 PMC4360138

[36] Boelens WC, Croes Y, de Jong WW. Interaction between alphaB-crystallin and the human 20S proteasomal subunit C8/alpha7. Biochim Biophys Acta. 2001;1544(1–2):311–9. doi: 10.1016/s0167-4838(00)00243-0 11341940

[37] Shen Z, Yang H, Yang S, Wang W, Cui X, Zhou X, et al. Hepatitis B virus persistence in mice reveals IL-21 and IL-33 as regulators of viral clearance. Nat Commun. 2017;8(1):2119. doi: 10.1038/s41467-017-02304-7 29242561 PMC5730569

[38] Shen Z, Wu J, Gao Z, Zhang S, Chen J, He J, et al. High mobility group AT-hook 1 (HMGA1) is an important positive regulator of hepatitis B virus (HBV) that is reciprocally upregulated by HBV X protein. Nucleic Acids Res. 2022;50(4):2157–71. doi: 10.1093/nar/gkac070 35137191 PMC8887475

[39] Kang N, Liu N, Liu M, Zhang S, Yang Y, Hou J, et al. Identification and characterization of host factor VCPIP1 as a multi-functional positive regulator of hepatitis B virus. J Virol. 2024;98(12):e0158124. doi: 10.1128/jvi.01581-24 39494904 PMC11650987

[40] Wei L, Shen Z, Zhao X, Wu Y, Liu W, Zhang J, et al. A broadly reactive monoclonal antibody detects multiple genotypes of hepatitis B virus X protein. Arch Virol. 2014;159(10):2731–5. doi: 10.1007/s00705-014-2111-6 24838854

[41] Tao S, Pan S, Gu C, Wei L, Kang N, Xie Y, et al. Characterization and engineering of broadly reactive monoclonal antibody against hepatitis B virus X protein that blocks its interaction with DDB1. Sci Rep. 2019;9(1):20323. doi: 10.1038/s41598-019-56819-8 31889135 PMC6937242

[42] Shen Z, Rao Y, Tao S, Luo M, Ming L, Liu J, et al. Unimpaired immunogenicity of yeast-expressed hepatitis B surface antigen stored at elevated temperatures. Acta Biochim Biophys Sin (Shanghai). 2016;48(12):1094–100. doi: 10.1093/abbs/gmw103 27827798

[43] Yuan H, Zhao L, Yuan Y, Yun H, Zheng W, Geng Y, et al. HBx represses WDR77 to enhance HBV replication by DDB1-mediated WDR77 degradation in the liver. Theranostics. 2021;11(17):8362–78. doi: 10.7150/thno.57531 34373747 PMC8343998

[44] Yang G, Feng J, Liu Y, Zhao M, Yuan Y, Yuan H, et al. HAT1 signaling confers to assembly and epigenetic regulation of HBV cccDNA minichromosome. Theranostics. 2019;9(24):7345–58. doi: 10.7150/thno.37173 31695772 PMC6831306

