# Supplementary figures and images for: Homeobox protein MSX-1 restricts hepatitis B virus by promoting ubiquitin-independent proteasomal degradation of HBx protein
Source: PLoS Pathog. 2025 Jan 30;21(1):e1012897. doi: 10.1371/journal.ppat.1012897 (PMC11781671; doi:10.1371/journal.ppat.1012897)

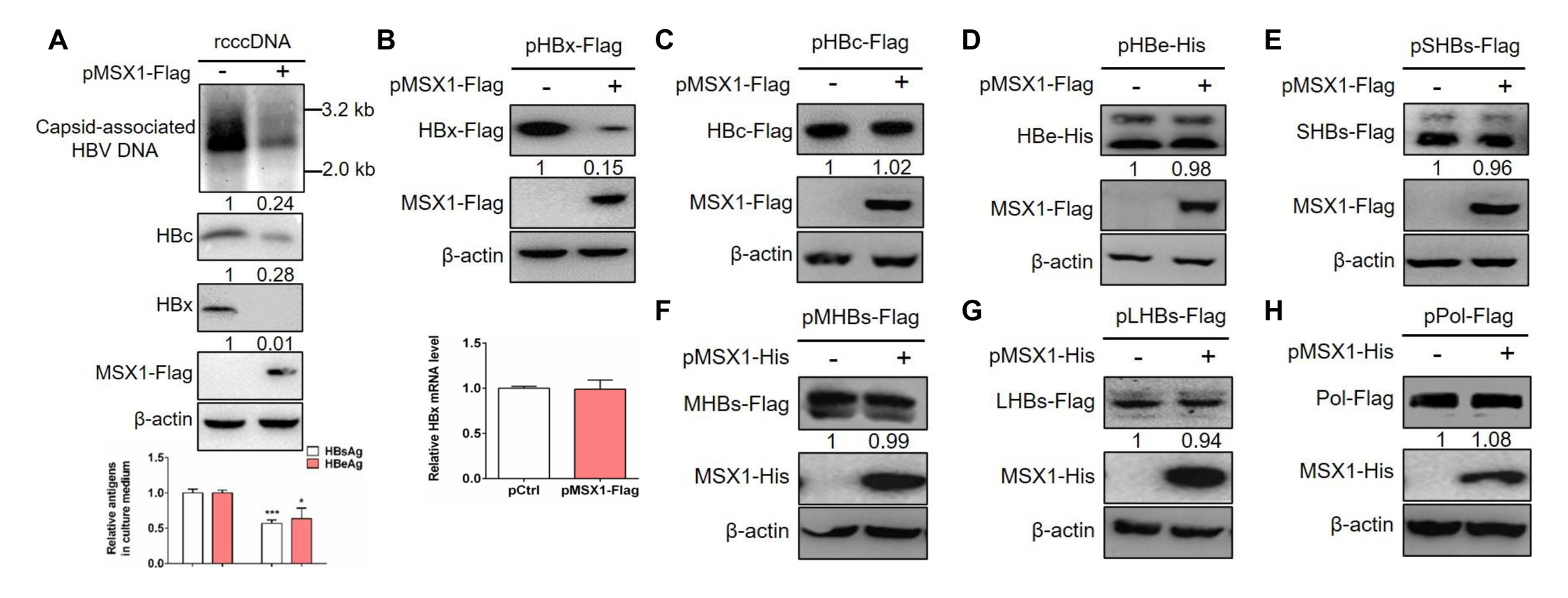

Supplement: S1 Fig — (A) Huh7 cells cultured in 6-well plates were transfected with 1μg of prcccDNA and 1 μg of pCre, plus 1μg of pMSX1-Flag or control plasmid (pCtrl). At 3 days post transfection, intracellular HBV replication and viral proteins (HBc and HBx) were assayed by Southern and Western blots, respectively. Exogenous MSX1 was determined in Western blot using Flag antibody and secreted antigens (HBsAg and HBeAg) were examined using ELISA. Huh7 cells cultured in 12-well plates were transfected with 0.5 μg of pHBx-Flag (B), pHBc-Flag (C), pHBe-His (D), pSHBs-Flag (E), pMHBs-Flag (F), pLHBs-Flag (G) or pPol-Flag (H), and 0.5 μg of pMSX1-Flag (B-E), pMSX1-His (F-H) or pCtrl. At 3 days post transfection, viral proteins and exogenous MSX1 were determined in Western blot using Flag or His antibody (B, top panel, C-H) while HBx mRNA levels determined in qrtPCR (B, bottom panel). HBV replication and protein levels were quantified using densitometry scanning and signal levels in control group were normalized as 1. Group means and SEMs of normalized values were presented and significances calculated using unpaired two-tailed t test. *, P < 0.05; ***, P < 0.001. (TIF) [file ppat.1012897.s001.tif]

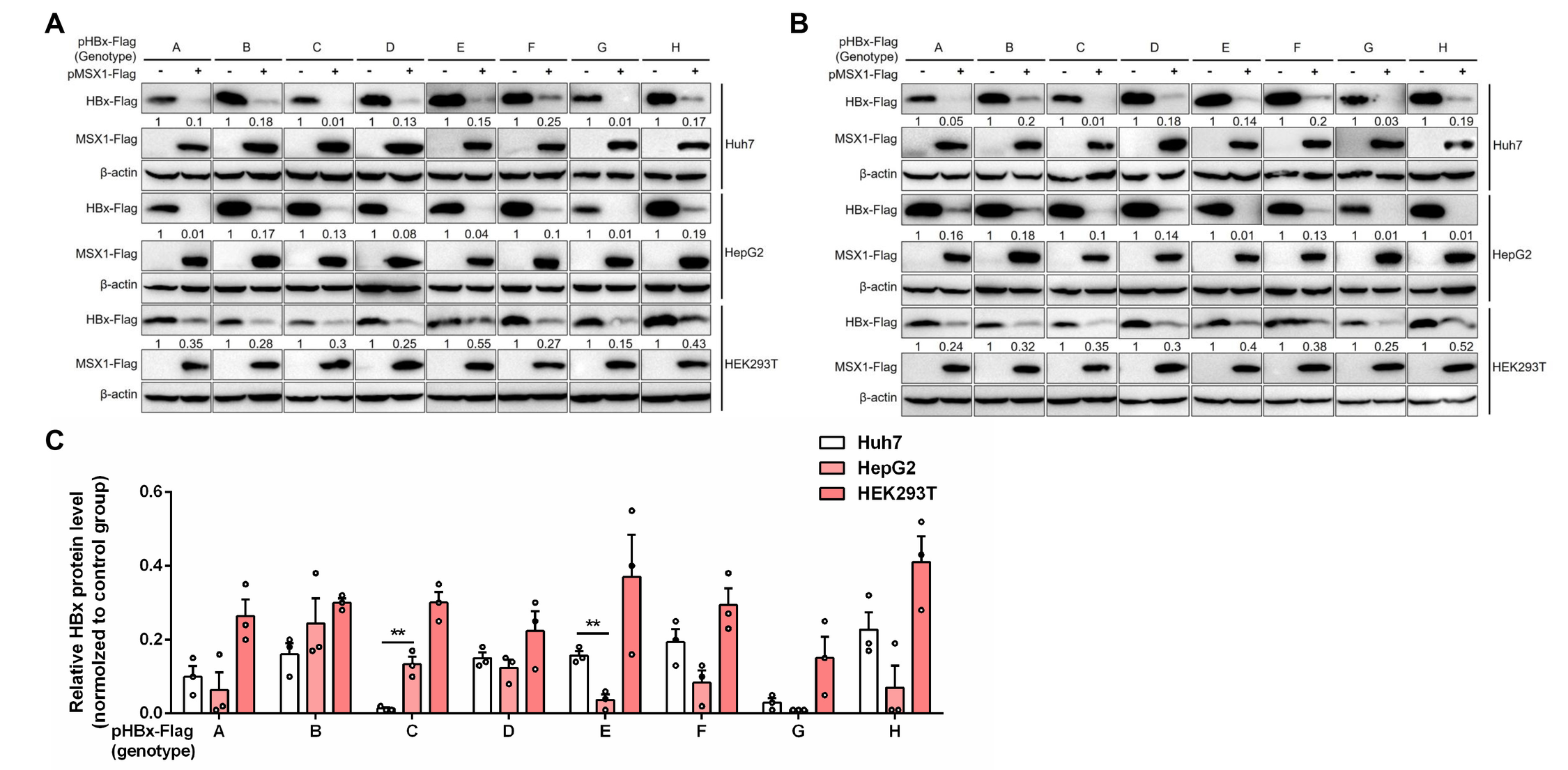

Supplement: S2 Fig — Huh7, HepG2 and HEK293T cells were transfected with pHBx-Flag of indicated HBV genotype and pMSX1-Flag at a transfection ratio of 1:1. At 3 days post transfection, HBx and exogenous MSX1 were determined in Western blot using Flag antibody. The experiments were repeated twice as shown in (A) and (B). HBx protein levels were quantified using densitometry scanning and signal levels in control group normalized as 1. (E) Analysis of MSX1’s effects on HBx protein expression of indicated genotype in hepatoma and HEK 293T cells based on three repeats (Figs 1D, S2A and S2B). HBx protein level in MSX1-transfected group was presented after normalized to control group. Group means and SEMs were presented and significances calculated using unpaired two-tailed t test. **, P < 0.01. (TIF) [file ppat.1012897.s002.tif]

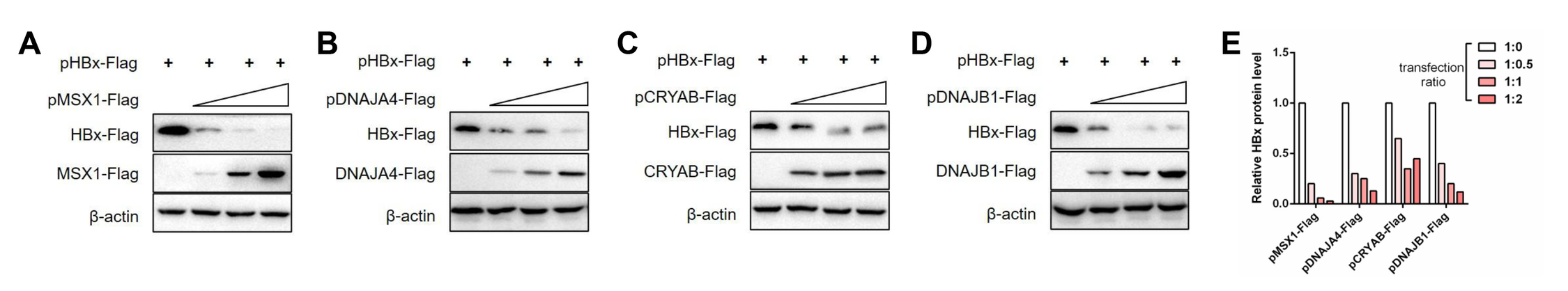

Supplement: S3 Fig — HepG2 cells were co-transfected with pHBx-Flag and an increasing amount of pMSX1-Flag (A), pDNAJA4-Flag (B), pCRYAB-Flag (C) or pDNAJB1-Flag (D) at a transfection ratio of 1:0, 1:0.5, 1:1, 1:2. At 3 days post transfection, the expression levels of HBx and exogenous genes were determined using Flag antibody in Western blot. (E) HBx protein levels were quantified using densitometry scanning and signal levels in control group were normalized as 1. (TIF) [file ppat.1012897.s003.tif]

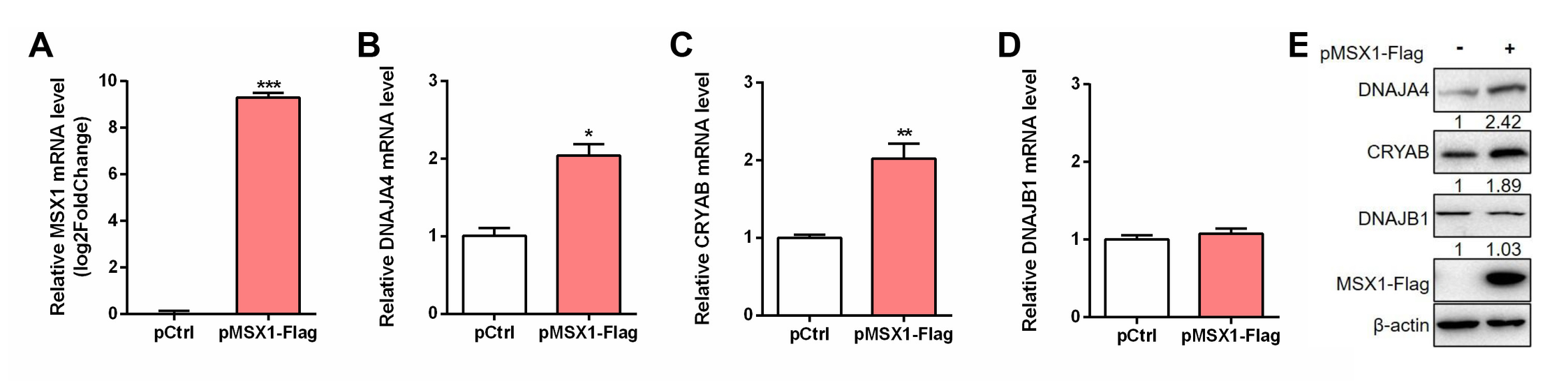

Supplement: S4 Fig — RT-qrtPCR (A-D) and Western blot assays (E) were performed on pMSX1-Flag- and pCtrl-transfected HepG2 cells to determine the effects of MSX1 on endogenous DNAJA4, CRYAB and DNAJB1 expression at mRNA and protein levels respectively. Protein levels were quantified using densitometry scanning and signal levels in control group were normalized as 1 (E). Group means and SEMs of normalized values were presented and significances calculated using unpaired two-tailed t test. *, P < 0.05; **, P < 0.01; ***, P < 0.001. (TIF) [file ppat.1012897.s004.tif]

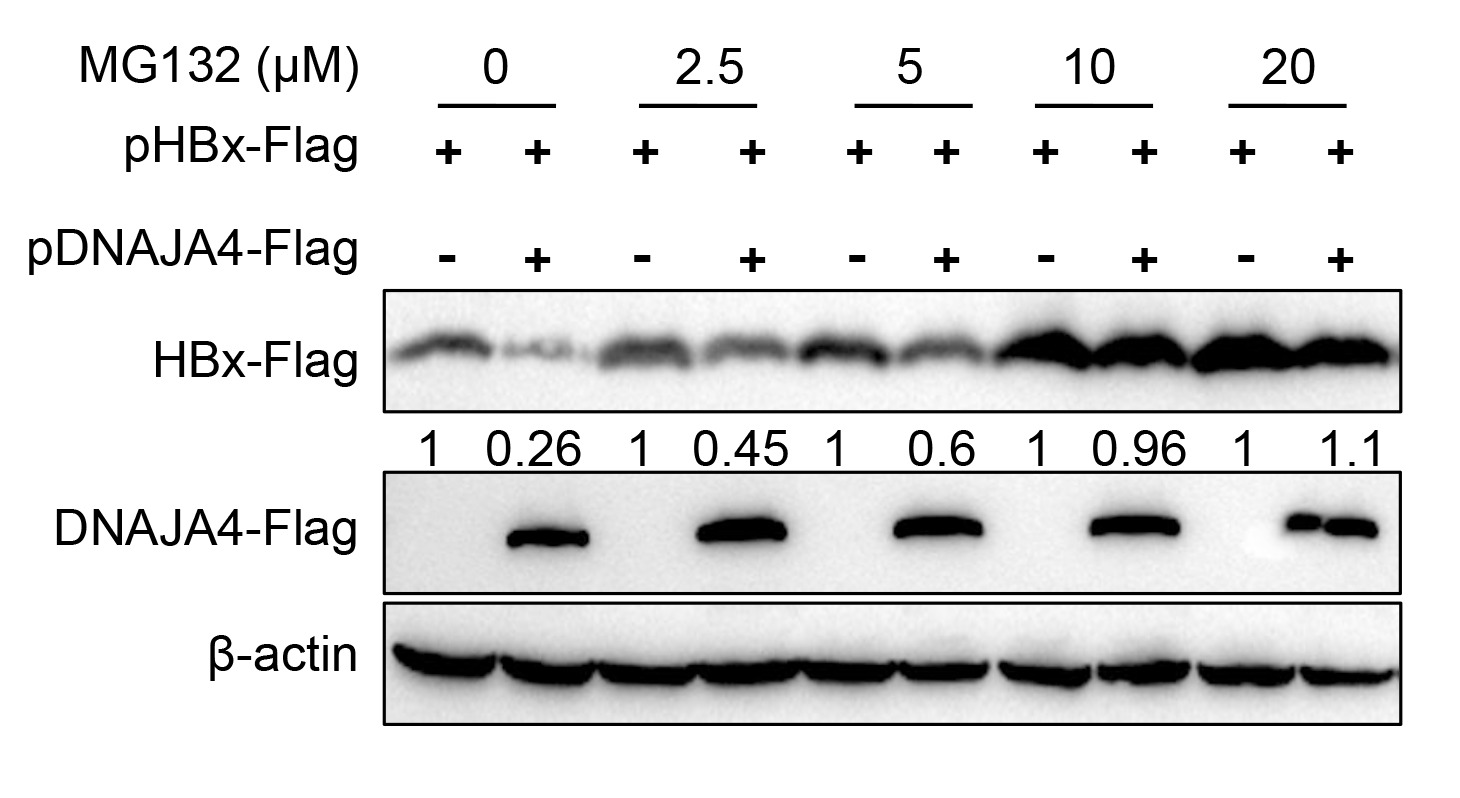

Supplement: S5 Fig — Huh7 cells were transfected with pHBx-Flag and pDNAJA4-Flag or pCtrl at a transfection ratio of 1:1. At 48 h post transfection, cells were treated with different concentrations of MG132 or left untreated for additional 24 h. HBx and exogenous DNAJA4 were determined in Western blot using Flag antibody. HBx protein levels were quantified using densitometry scanning and signal levels in pCtrl-transfected group were normalized as 1. (TIF) [file ppat.1012897.s005.tif]

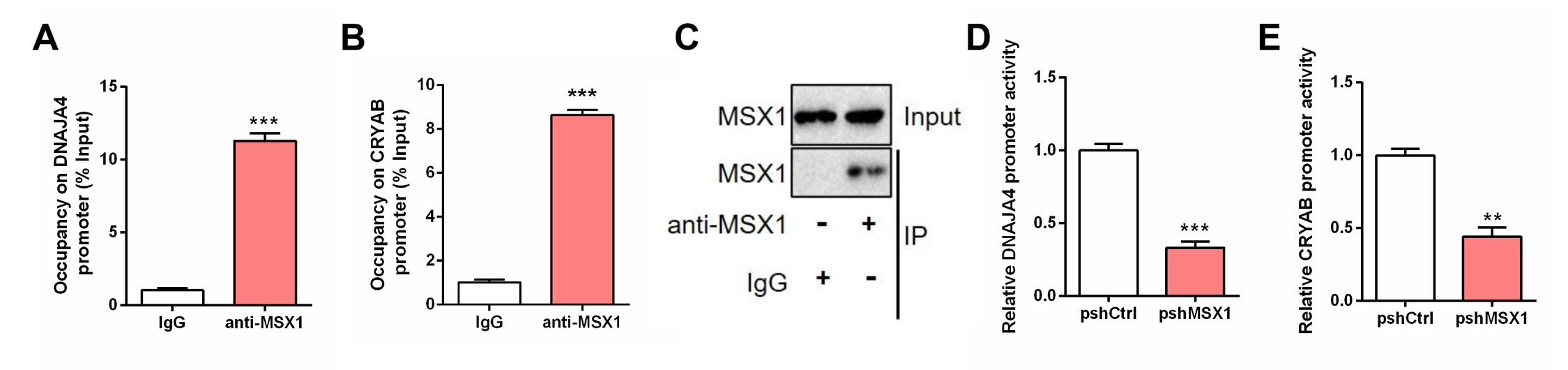

Supplement: S6 Fig — Huh7 cells were subjected to ChIP assay, and sonicated DNA immunoprecipitated by anti-MSX1 or rabbit control IgG was quantitated in qrtPCR using specific primers targeting DNAJA4 promoter (A) or CRYAB promoter (B) and indicated as percentage of input. Immunoprecipitated MSX1 was determined in Western blot (C). Huh7 cells cultured in 24-well plates were transfected with 0.3 μg of DNAJA4 promoter reporter plasmid (D) or CRYAB promoter reporter plasmid (E), plus 0.1 μg of pRL-TK and 0.3 μg of pshMSX1 or pshCtrl. At 2 days post transfection, cells were lysed and promoter activities were measured using dual-luciferase reporter assay. Group means and SEMs of normalized values were presented and significances calculated using unpaired two-tailed t test. **, P < 0.01; ***, P < 0.001. (TIF) [file ppat.1012897.s006.tif]

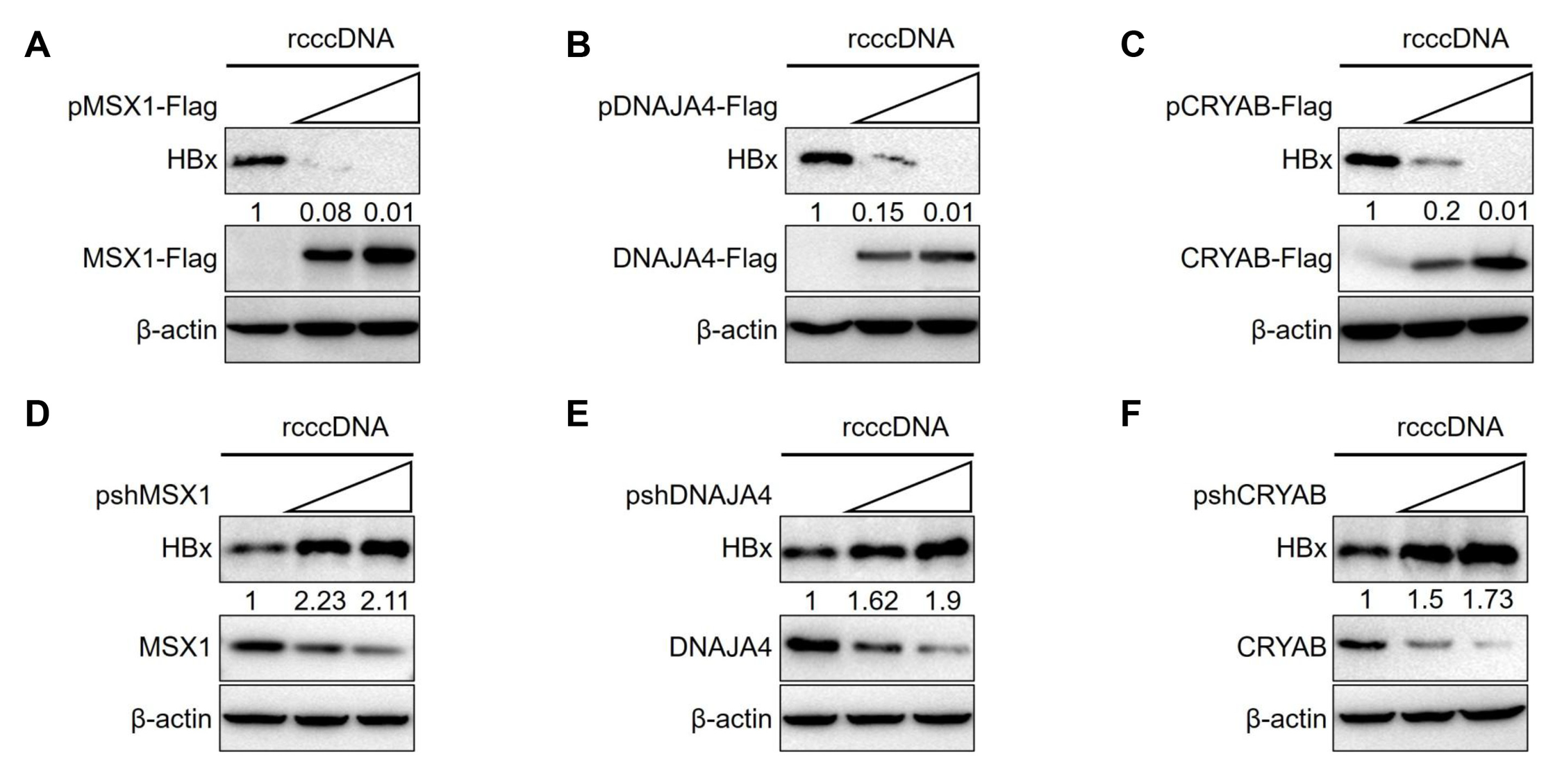

Supplement: S7 Fig — Huh7 cells cultured in 12-well plates were transfected with 0.5 μg of prcccDNA and 0.5 μg of pCre, plus 0.1 or 0.5 μg of pMSX1-Flag (A), pDNAJA4-Flag (B) or pCRYAB-Flag (C), or plus 0.25 or 0.5 μg of pshMSX1 (D), pshDNAJA4 (E) or pshCRYAB (F). At 3 days post transfection, HBx, exogenous and endogenous MSX1, DNAJA4 and CRYAB were determined using Western blot. HBx protein levels were quantified using densitometry scanning and signal levels in control group were normalized as 1. (TIF) [file ppat.1012897.s007.tif]

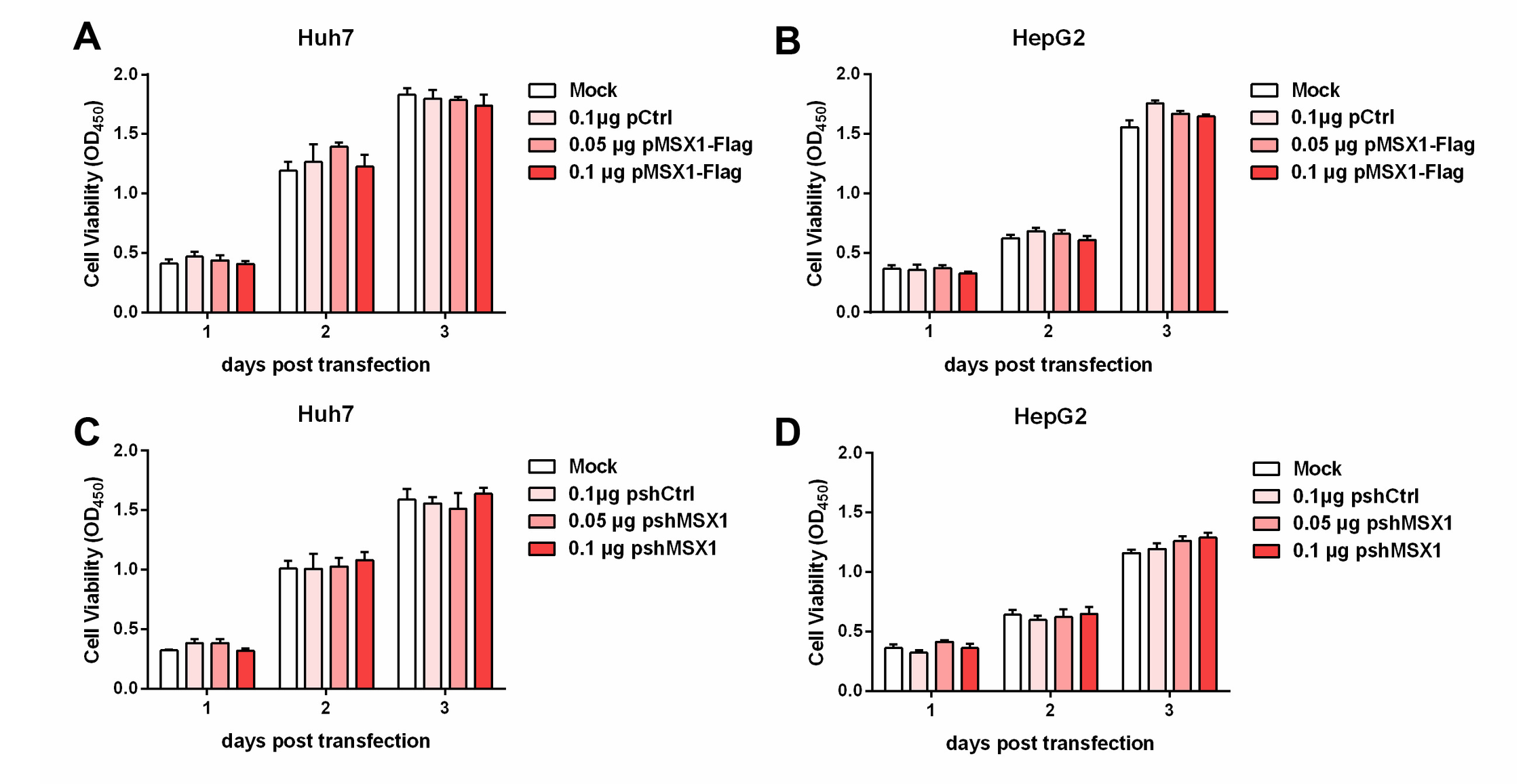

Supplement: S8 Fig — Hepatoma cells (Huh7 and HepG2) cultured in 96-well plates were transfected with indicated amounts of pMSX1-Flag or pCtrl (A-B), pshMSX1 or pshCtrl (C-D), or left untreated. Cell viability was detected at day 1, 2 and 3 post transfection using CCK-8 assays. Group means and SEMs within transfection group were presented. (TIF) [file ppat.1012897.s008.tif]

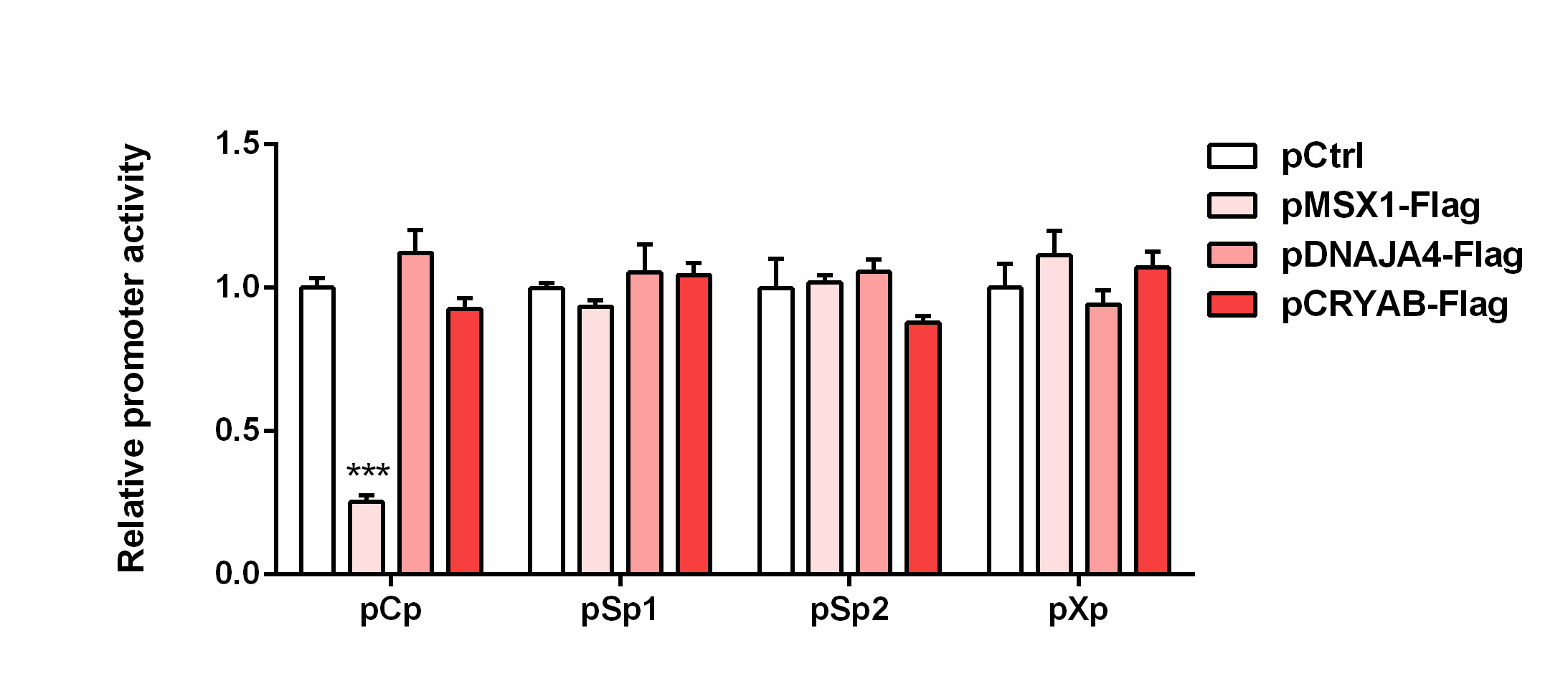

Supplement: S9 Fig — Huh7 cells cultured in 24-well plates were transfected with 0.3 µg of Cp, Sp1, Sp2, or Xp reporter plasmid, 0.3 µg of pMSX1-Flag, pDNAJA4-Flag, pCRYAB-Flag or pCtrl, and 0.1 µg of pRL-TK. At 48 h post transfection, cells were lysed and subjected to dual-luciferase reporter assay. Group means and SEMs of normalized firefly versus Renilla luciferase activity ratios were presented, and significances calculated using unpaired two-tailed t-test. ***, P < 0.001. (TIF) [file ppat.1012897.s009.tif]

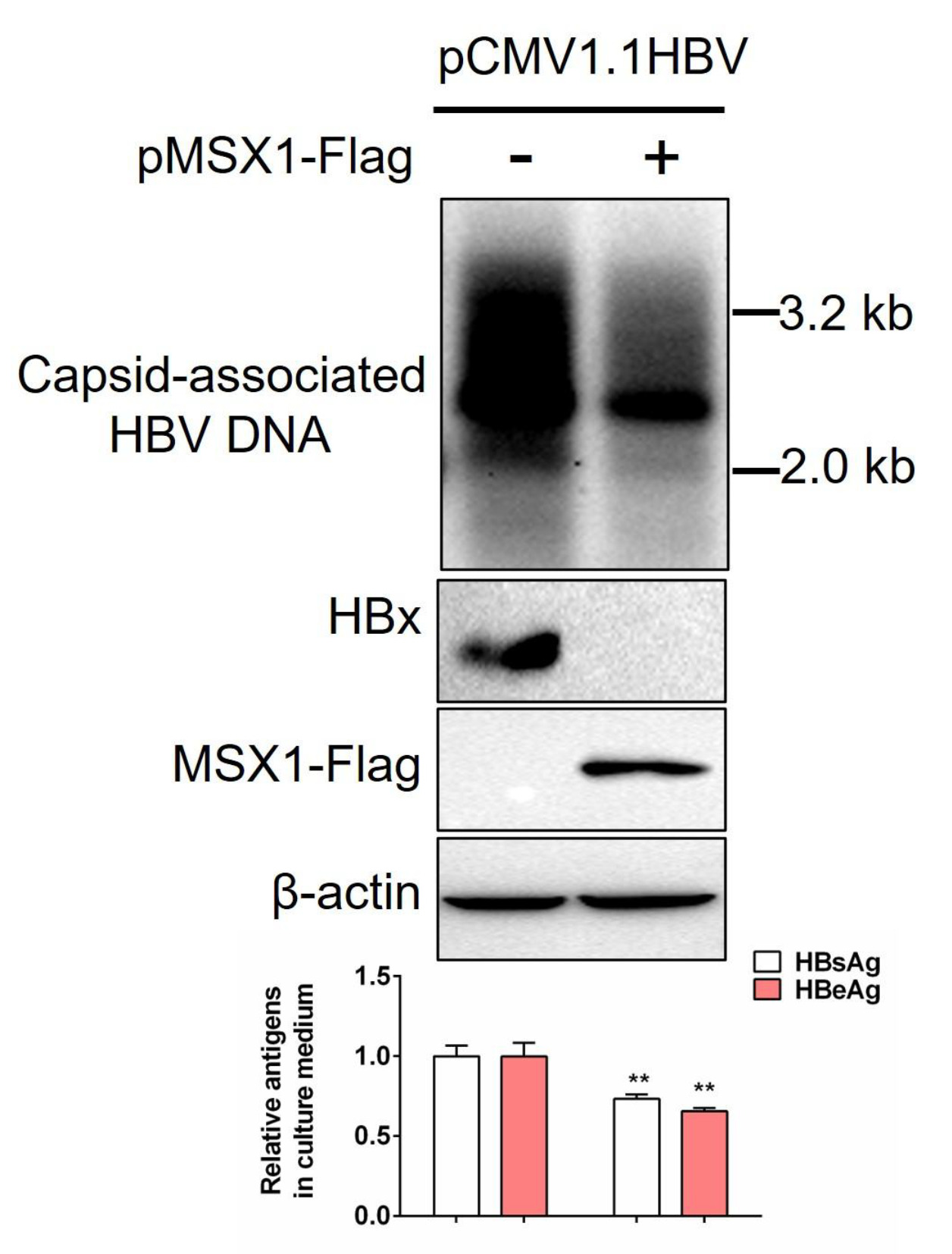

Supplement: S10 Fig — Huh7 cells cultured in 12-well plates were transfected with 1 µg of pCMV1.1HBV and 1 µg of pMSX1-Flag or pCtrl. At 3 days post transfection, intracellular HBV replication, HBx and exogenous MSX1 were analyzed using Southern and Western blots, respectively. Secreted HBV antigens (HBsAg and HBeAg) were assayed using ELISA. Group means and SEMs of normalized values were presented and significances calculated using unpaired two-tailed t test. **, P < 0.01. (TIF) [file ppat.1012897.s010.tif]

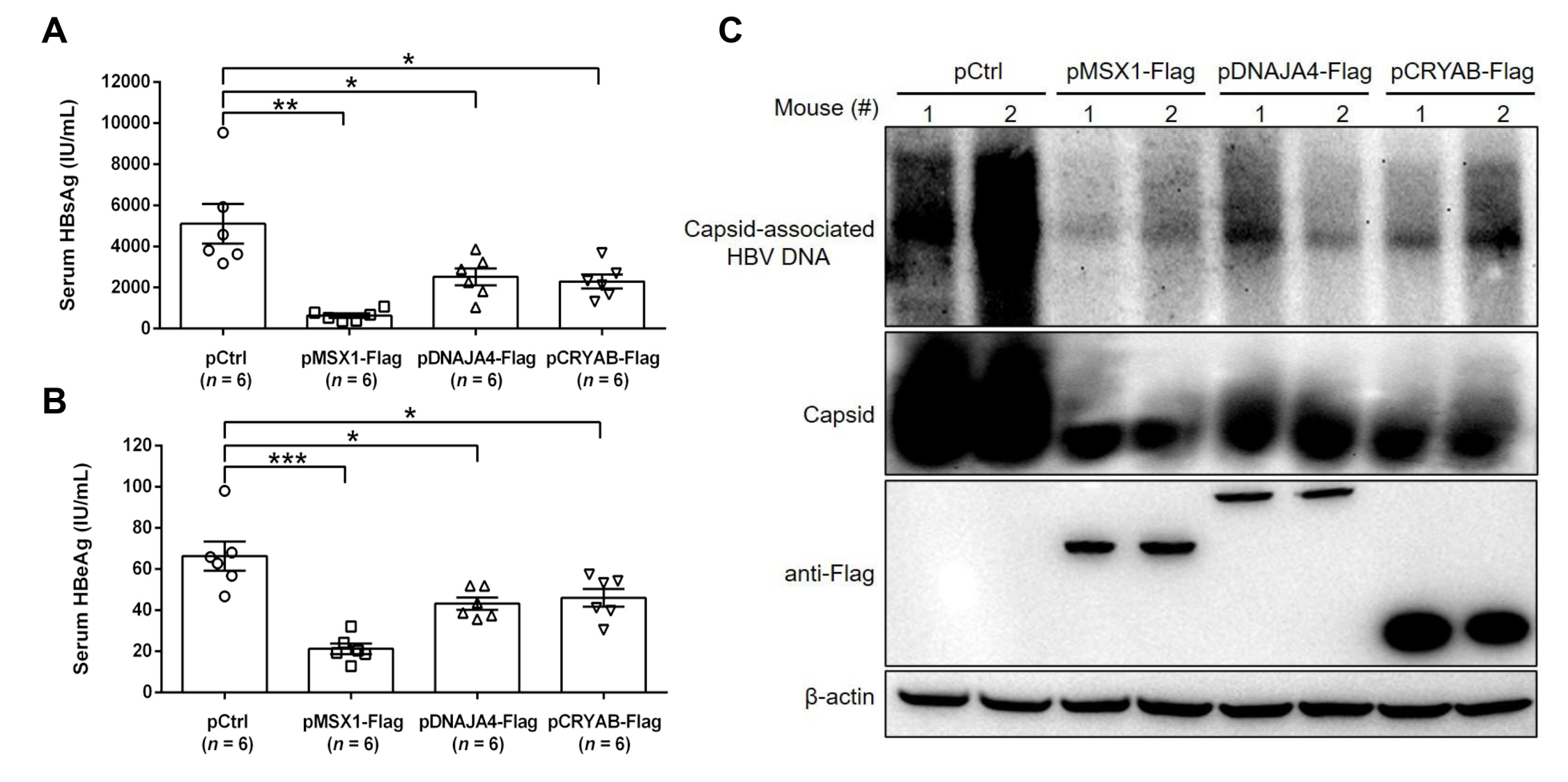

Supplement: S11 Fig — BALB/c mice were co-injected with prcccDNA and pCre, plus pMSX1-Flag, pDNAJA4-Flag, pCRYAB-Flag or pCtrl through HDI method. At 3 days post injection, serum and liver tissues samples were taken. Serum HBsAg (A) and HBeAg (B) were analyzed using commercial quantitative assays with group size indicated (n). (C) Intracellular HBV replication and exogenous genes expression were analyzed using Southern and Western blots, respectively. Capsid levels were determined using native agarose gel electrophoresis followed by Western blot. Group means and SEMs were presented and significances calculated using unpaired two-tailed t test. *, P < 0.05; **, P < 0.01; ***, P < 0.001. (TIF) [file ppat.1012897.s011.tif]

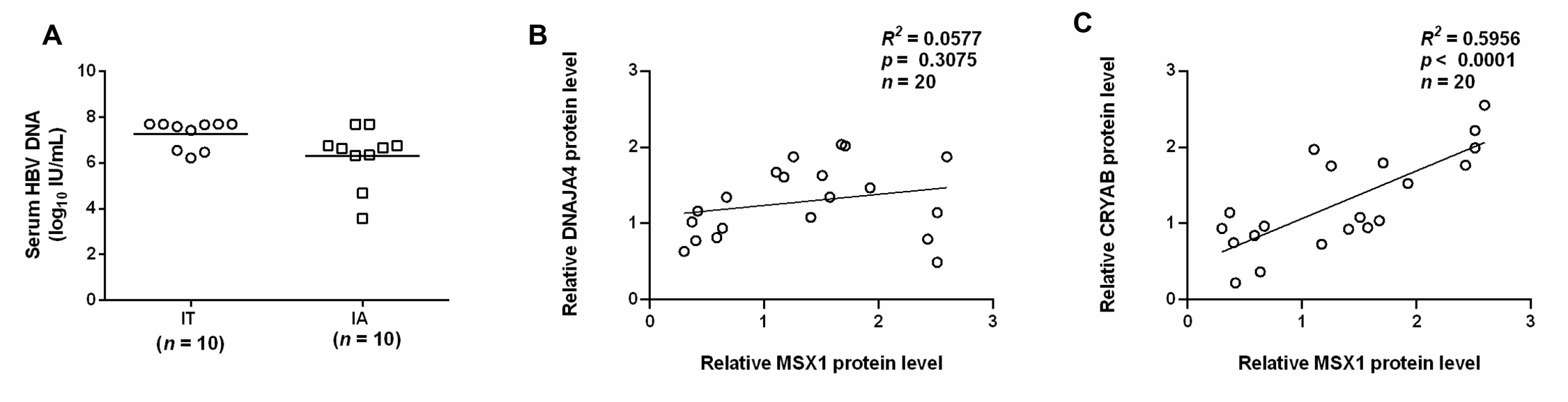

Supplement: S12 Fig — (A) Comparison of serum HBV DNA between IT and IA phases with group size (n) indicated. The correlation analysis between MSX1 and DNAJA4 (B) or CRYAB (C) at protein expression levels based on the data from Fig 7E, 7F, 7G and 7I. (TIF) [file ppat.1012897.s012.tif]

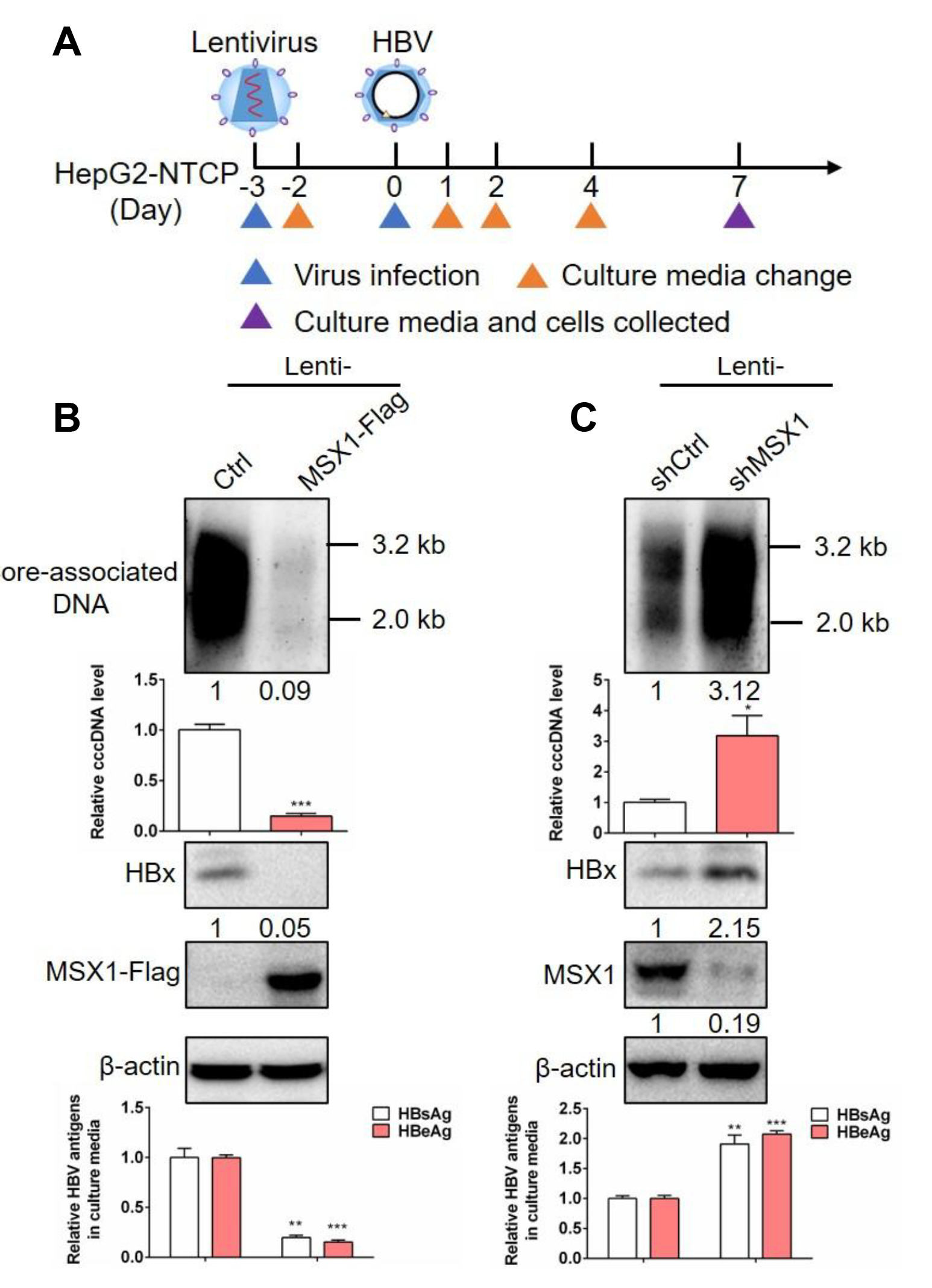

Supplement: S13 Fig — HepG2-NTCP cells were first transduced with Lenti-MSX1-Flag or Lenti-Ctrl (A and B), or Lenti-shMSX1 or Lenti-shCtrl (A and C), and 3 days later, infected with HBV at 1000 geq/cell. Culture media were changed at indicated time points. At 7 days post HBV infection, culture media and cells were collected for further analysis. HBsAg and HBeAg in culture media were analyzed using ELISA. Intracellular HBV replication and cccDNA were analyzed using Southern blot and qrtPCR, respectively. HBx and exogenous/endogenous MSX1 were analyzed using Western blot. Viral replication and protein levels were quantified using densitometry scanning and signal levels in control group were normalized as 1. Group means and SEMs were presented and significances calculated using unpaired two-tailed t test. *, P < 0.05; **, P < 0.01; ***, P < 0.001. (TIF) [file ppat.1012897.s013.tif]

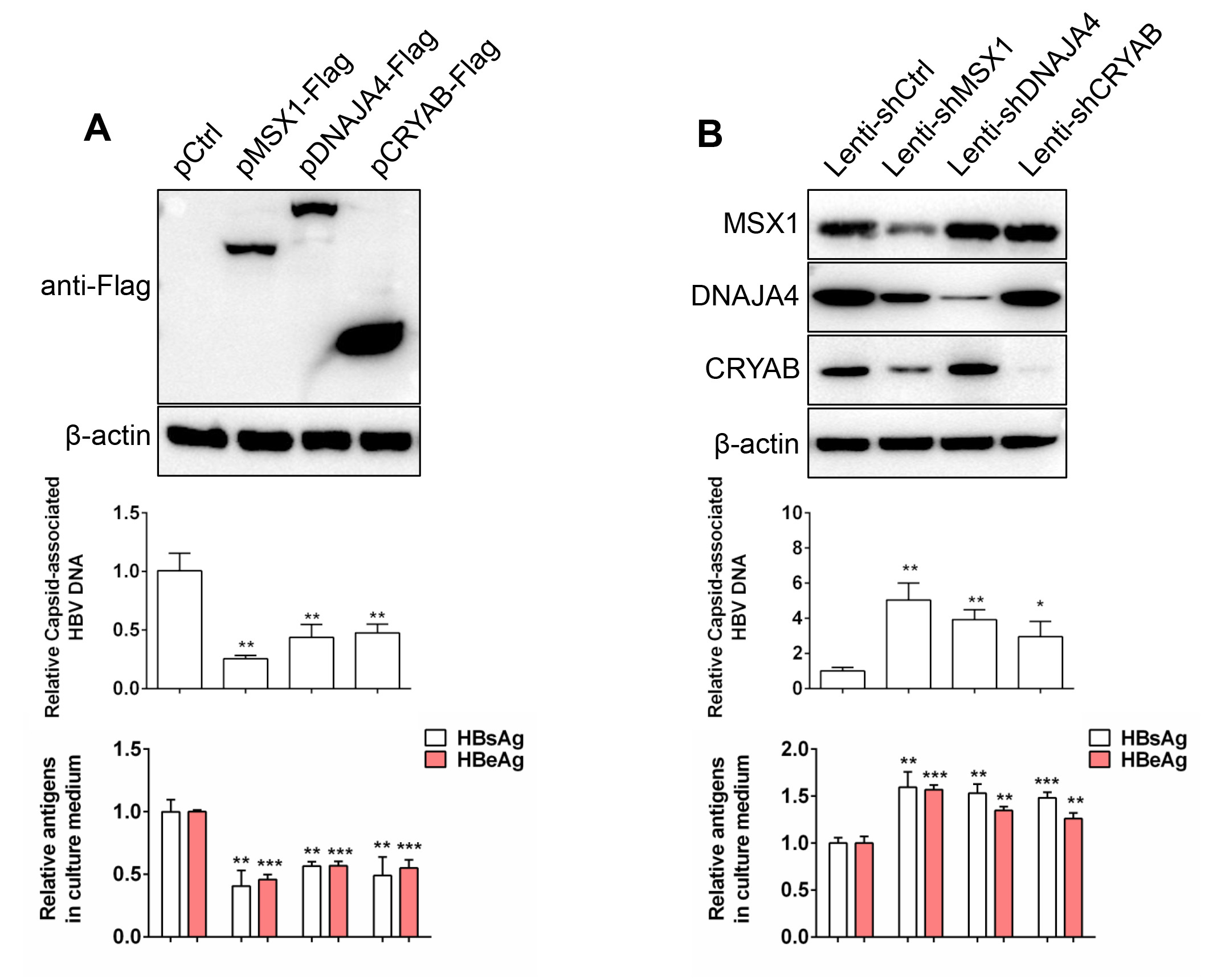

Supplement: S14 Fig — HepG2-NTCP cells in 24-well pates were transfected with 1 μg of pMSX1-Flag, pDNAJA4-Flag, pCRYAB-Flag or pCtrl (A), or transduced with Lenti-shMSX1, Lenti-DNAJA4, Lenti-CRYAB or Lenti-shCtrl (B), and 3 days later, infected with HBV at 1000 geq/cell. Culture media were changed at day 1, 2 and 4 post HBV infection. At 7 days post HBV infection, HBsAg and HBeAg in culture media were analyzed using ELISA. Intracellular HBV replication was analyzed using qrtPCR. Exogenous/endogenous MSX1, DNAJA4 and CRYAB were analyzed using Western blot. Viral replication and serum antigens in control group were normalized as 1. Group means and SEMs were presented and significances calculated using unpaired two-tailed t test. *, P < 0.05; **, P < 0.01; ***, P < 0.001. (TIF) [file ppat.1012897.s014.tif]
