# Supplementary material for: Homeobox protein MSX-1 restricts hepatitis B virus by promoting ubiquitin-independent proteasomal degradation of HBx protein
Source: PLoS Pathog. 2025 Jan 30;21(1):e1012897. doi: 10.1371/journal.ppat.1012897 (PMC11781671; doi:10.1371/journal.ppat.1012897)
Supplement: S2 Table — (DOCX) [file ppat.1012897.s016.docx]

**S2 Table.** Clinical and virological information of CHB patients in IT and IA phases

|  | Immune tolerant (*n* = 10) | Immune active (*n* = 10) |
| --- | --- | --- |
| Age (yrs)* | 29 (23-34) | 40 (25-60) |
| Gender (M/F) | 5/5 | 6/4 |
| HBV DNA  (log_10_ IU/mL) | 7.27 (6.22-7.69) | 6.31 (3.57-7.69) |
| HBsAg(+/-) | 10/0 | 10/0 |
| HBeAg(+/-) | 10/0 | 10/0 |
| ALT (U/L)* | 28 (15-34) | 185 (71-1232) |
| Inflammation  G0 / G1 / G2 / G3 / G4, *n* | 5/4/1/0/0 | 0/2/4/4/2 |
| Fibrosis  S0 / S1 / S2 / S3 / S4, *n* | 3/5/2/0/0 | 0/1/5/2/2 |

* expressed as X ± SD. Inflammation and fibrosis were graded using blinded liver biopsy sections by qualified pathologists according to the standards of Scheuer System.
