# Supplementary material for: Homeobox protein MSX-1 restricts hepatitis B virus by promoting ubiquitin-independent proteasomal degradation of HBx protein
Source: PLoS Pathog. 2025 Jan 30;21(1):e1012897. doi: 10.1371/journal.ppat.1012897 (PMC11781671; doi:10.1371/journal.ppat.1012897)
Supplement: S3 Table — (DOCX) [file ppat.1012897.s017.docx]

**S3 Table.** Primer sequences used for RT-qrtPCR or qrtPCR analysis

| Target | Primer | Sequence |
| --- | --- | --- |
| MSX1 | Forward | gacgcccttcaccaccgcgc |
|  | Reverse | TCCAGCTCTGCCTCTTGTAG |
| HSPA6 | Forward | CAAGGTGCGCGTATGCTAC |
|  | Reverse | GCTCATTGATGATCCGCAACAC |
| HSPA1A | Forward | TTCGGAGAGTTCTGGGATTGTA |
|  | Reverse | TGGACTGTTCTTCACTCTTGGC |
| DNAJA4 | Forward | ATGACATCCTGGGCGTGAAG |
|  | Reverse | CCTCATCCGGGTTCTTGTCC |
| DNAJB1 | Forward | GACCCATTCTCTGGCTTCCC |
|  | Reverse | TCTTTCCGTCGGGGTTTAGC |
| HSPA1B | Forward | CACCGTGTTTGACGCGAAGC |
|  | Reverse | TGGGCTTGTCTCCGTCGTTGA |
| VGF | Forward | GGAACTGCGAGATTTCAGTCC |
|  | Reverse | GTGCGGGTTTCCGTCTCTG |
| SOX8 | Forward | AGAAGGACCACCCCGACTAC |
|  | Reverse | AGCCCTGCTTCAGCCTTGTA |
| CRYAB | Forward | CTTTGACCAGTTCTTCGGAG |
|  | Reverse | CCTCAATCACATCTCCCAAC |
| RRAD | Forward | aggcagcagggcacacctat |
|  | Reverse | tcgaagctgcccttgtccgt |
| β-actin | Forward | AAGGTGACAGCAGTCGGTT |
|  | Reverse | TGTGTGGACTTGGGAGAGG |
| DNAJA4 promoter | Forward | ACTGTCGGCCCTTGGCAGAC |
|  | Reverse | CTCATGACTGCAAGGCTGAG |
| CRYAB promoter | Forward | CCCGGAGAGCCAGGGCTCGA |
|  | Reverse | CTCAGCTGTCCTCTCAGGCC |
| HBx and | Forward | ACCGACCTTGAGGCATACTT |
| HBV total RNA | Reverse | GCCTACAGCCTCCTAGTACA |
| HBV 3.5 kb RNA and | Forward | AATGCCCCTATCTTATCAACACT |
| Capsid-associated DNA | Reverse | GAGATTGAGATCTTCTGCGACG |
| cccDNA | Forward | TGCACTTCGCTTCACCT |
|  | Reverse | AGGGGCATTTGGTGGTC |
